# Impact of Side-Chain Polarity and Symmetry on the Structure and Properties of Acyclic Dioxythiophene Polymers

**DOI:** 10.1021/acs.chemmater.4c03394

**Published:** 2025-06-20

**Authors:** Joshua M. Rinehart, Zhuang Xu, Ziming Wang, Anna M. Österholm, Lucas Q. Flagg, Lee J. Richter, Chad R. Snyder, Ying Diao, John R. Reynolds

**Affiliations:** † School of Materials Science and Engineering, 1372 Georgia Institute of Technology, Atlanta, Georgia 30332, United States; Department of Chemical and Biomolecular Engineering, Department of Chemistry, Beckman Institute for Advanced Science and Technology, 14589 University of Illinois Urbana–Champaign, Urbana, Illinois 61801, United States; § School of Chemistry and Biochemistry, Georgia Institute of Technology, Atlanta, Georgia 30332, United States; ∥ 96988 National Institute of Standards and Technology, Gaithersburg, Maryland 20899, United States

## Abstract

Conformationally flexible side-chains on conjugated polymers promote solution processability while significantly impacting solution aggregation, solid-state ordering, and polymer–electrolyte interactions. These side-chains can strongly influence the repeat unit symmetry, polarity, steric bulk, and noncovalent interactions that collectively dictate how polymer chains pack and assemble. Acyclic dioxythiophene polymers (PAcDOTs) are highly redox-active thiophene-based organic mixed ionic-electronic conductors (OMIECs) where both the 3- and 4-positions of the thiophene ring are substituted by alkoxy groups. In this study, we explore the solution and solid-state structures of both symmetric (R1 = R2) and asymmetric (R1 ≠ R2) PAcDOTs, where R1 and R2 are both either nonpolar or amphiphilic, and we observe rich process-structure behavior. The combination of regio-symmetric and amphiphilic side-chains results in a chiral, lyotropic phase in chloroform solutions that persists as a metastable state in thin films. Temperature-dependent grazing incidence X-ray scattering and cross-polarized optical microscopy indicate that PAcDOTs with regio-symmetric linear side-chains can crystallize into highly ordered morphologies upon thermal treatment. Finally, we show that side-chain polarity, symmetry, and thermal annealing affect the electrochemical doping and dedoping processes.

## Graphical abstract

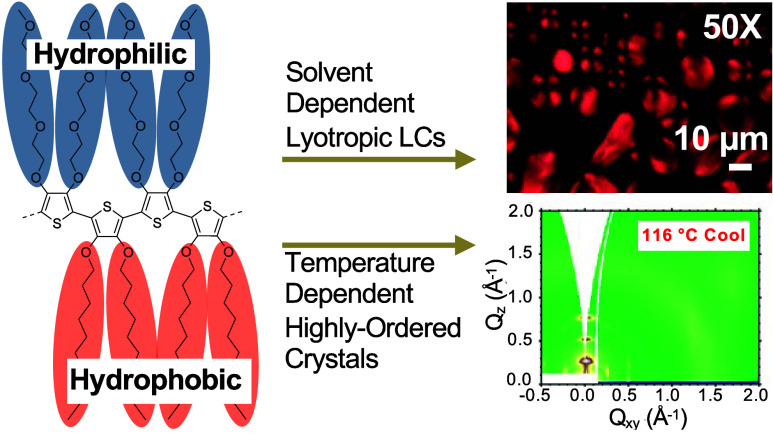

## Introduction

Polythiophene and its derivatives have become foundational structures in the field of conjugated polymers with the thiophene unit serving as a versatile building block for the design of new polymers with diverse structures and properties.^1–3^ Among these, poly­(3-hexylthiophene) (P3HT) and poly­(3,4-ethylenedioxythiophene) (PEDOT) have stood out over decades owing to their widespread availability and proven effectiveness in various electronic and photovoltaic applications. For example, P3HT and other poly­(3-alkylthiophenes) (P3ATs) exhibit respectable hole mobility, making them prime materials for organic field effect transistors (OFETs) and organic photovoltaics.^4–6^ However, to optimize hole mobility, P3ATs must be made highly regioregular (RR), which complicates polymer synthesis by requiring specific conditions and catalysts that are not suitable for all thiophene derivatives.^7,8^

The material properties of P3HT are strongly correlated to the degree of regioregularity, and consequently, sample-to-sample variations yield drastically different processing behaviors and material properties, ultimately yielding devices with processing-dependent properties.^9–13^ Head-to-tail regioregularity produces P3ATs with the most planar backbones and lowest bandgaps compared to regiorandom or head-to-head regiochemistry.^14^ For large-scale synthesis and potential commercialization, it is desirable to have polymers whose structure and subsequent properties remain consistent without the need for highly controlled synthetic conditions to achieve high levels of regioregularity. This can be accomplished by designing monomers and repeat units with inherent symmetry to ensure the formation of regio-symmetric repeat units.

Designing monomers with a *C*_2_ symmetry axis yields symmetric repeat units, enabling simpler polymerization conditions for generating regio-symmetric polymers. Figure 1 illustrates how symmetric monomers lead to regio-symmetric repeat units because all coupling modes between thiophene units—2,2′, 2,5′, 5,2′, and 5,5′—are equivalent. In contrast, asymmetric side-chain substitution, such as in P3ATs, introduces chemically distinct regiochemical linkages (e.g., 2,2′ vs 2,5′ linkages), thereby disrupting regio-symmetry.^15^

**Figure 1:**
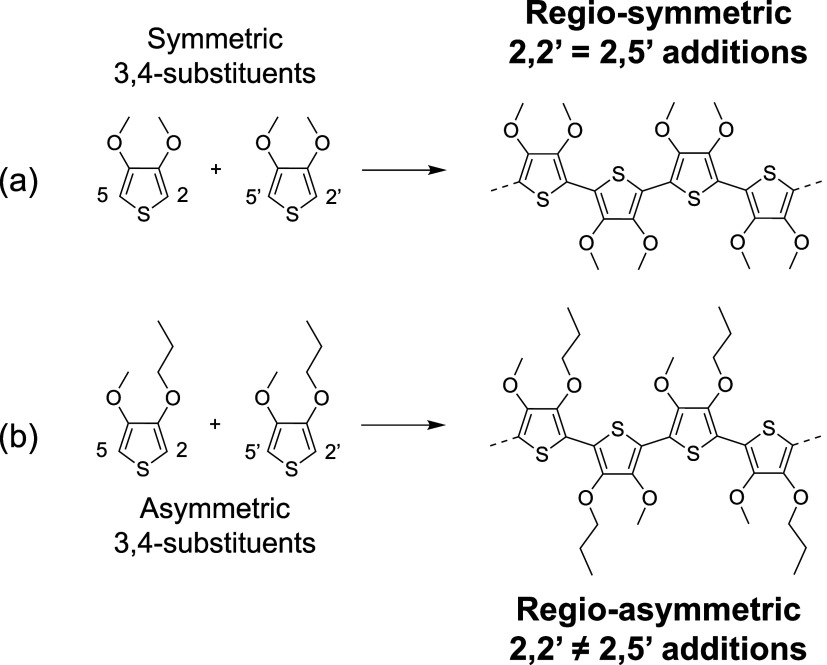
(a) Symmetric side-chain substitution enables regio-symmetric repeat units as all possible regiochemical linkages are equivalent. (b) Asymmetric side-chain substitution, in this case for a theoretical 3-methoxy-4-propoxythiophene polymer, results in nonequivalent 2,2′ and 2,5′ linkages, introducing disorder along the polymer backbone. A specialized polymerization method that is selective for one regiochemical linkage is necessary for regioregularity.

One of the most recognizable regio-symmetric conjugated polymers is poly­[2,5-bis­(3-tetradecylthiophen-2-yl)­thieno­[3,2-*b*]­thiophene], commonly referred to as pBTTT-C14.^16–18^ The side-chain substitution pattern, tilt of the linear alkyl chains, and a regio-symmetric repeat unit result in multiple thermal phase transitions. Depending on processing, OFET hole mobilities can exceed 0.5 cm^2^ V^–1^ s^–1^ and electrical conductivities can surpass 1000 S cm^–1^ when chemically doped.^19,20^ Another example of how regio-symmetry can be used to induce long-range order has been demonstrated with thiophene-phenylene-thiophene (TPT) polymers by modifying the side-chains on the phenylene ring.^21,22^ Symmetrically placed side-chains result in highly ordered materials with π-π and lamellar stacking, while asymmetrically placed side-chains (regio-asymmetric repeat unit) result in mostly amorphous scattering.^21^

In addition to regio-symmetry, side-chain amphiphilicity can induce unique morphologies and modulate properties in P3ATs and other conjugated polymers.^23–26^ Amphiphilic polythiophenes can assemble at the air–water interface and form well-ordered 2D crystals, capable of rotating light in cross-polarized optical microscopy (CPOM).^24^ Upon melting and cooling, a polythiophene bearing a decyl-tri­(oxyethylene) side-chain resulted in what appeared to be a liquid crystalline phase that persisted until near room temperature.^25^

Here, we report a family of 3,4-disubstituted acyclic dioxythiophene polymers (PAcDOTs) with either regio-symmetric or regio-asymmetric repeat units bearing alkyl and/or oligoether side-chains. Previous work on PAcDOTs has shown that subtle changes in side-chain structure—such as linear versus branched alkoxy groups—can lead to profound differences in solid-state order and redox behavior.^27–29^ These polymers offer synthetic scalability and tunability, enabling control over side-chain polarity and symmetry, both of which heavily influence polymer–polymer and polymer–solvent interactions. As with other dioxythiophene polymers, electron donation into the thiophene ring lowers oxidation potential and improves many redox properties, while noncovalent S–O interactions can lead to higher barriers for dihedral bond rotation, helping to lock in certain chain conformations.^22,30–32^ We also detail a streamlined, high-yield process for synthesizing large quantities of 3,4-bis­(2-(2-(2-methoxyethoxy)­ethoxy)­ethoxy)­thiophene (AcDOT-[OE_3_]_2_) monomer with high purity in a single step from commercially available 3,4-dimethoxythiophene, facilitating scalable access to AcDOT-based polymers. The AcDOT monomers and polymers were prepared at scale, and the relatively simple repeat units allowed us to probe how side-chain polarity and regio-symmetry impact solution-state aggregation, solid-state ordering, as well as electrochemical properties.

Regio-symmetric, amphiphilic PAcDOT-[C_10_]_2_-*co*-[OE_3_]_2_ can form chiral, lyotropic liquid crystals (LCs) in chloroform (CHCl_3_) solutions that persist as a metastable, chiral mesophase after blade coating. Differential scanning calorimetry (DSC) indicates that PAcDOTs have a backbone melt transition, and temperature-dependent grazing incidence wide-angle X-ray scattering (GIWAXS) gives evidence of highly ordered crystalline phases when cooled from the melt. While regio-symmetric PAcDOTs display unique ordering and phase transitions, the high degree of side-chain order in PAcDOT-[C_10_]_2_ inhibits the doping and dedoping process. Despite lacking symmetry, regio-asymmetric PAcDOTs have a clear backbone melting transition and order after thermal annealing, in contrast to regiorandom polythiophenes.^9^ Despite all having the same conjugated backbone, the side-chain design heavily influences the solution morphology, solid-state crystallinity, and mixed ionic-electronic transport.

## Experimental Section

### Materials and Methods

Detailed information, including monomer synthesis, polymer synthesis, polymer characterization, GIWAXS, electrochemistry, CPOM, circular dichroism spectroscopy (CD), and instrumentation, is available in the Supporting Information.

### Film Preparation and Annealing

All substrates were cleaned by sonication in sodium dodecylbenzenesulfonate aqueous solutions, followed by DI water and finally isopropanol. Films were blade-coated from room temperature 15 mg/mL CHCl_3_ solutions with a Zehntner Testing Instruments blade coater (a ZAA 2300 equipped with a ZUA 2000 blade). A blade height of 150 μm above the substrate and speed of 40 mm/s was used. Films were coated twice to achieve optical absorbance near 1 (thicknesses ranging between 150 and 250 nm).

Films labeled as annealed were heated to approximately 10 °C above the melting temperature (160 °C for regio-symmetric polymers and 240 °C for regio-asymmetric polymers) on a hot plate inside a glovebox with argon atmosphere, then slowly cooled to room temperature at approximately 3 °C/min.

## Results and Discussion

### Monomer and Polymer Synthesis and Characterization

Monomers with disubstituted alkoxy side-chains were prepared by acid-catalyzed transetherification, similar to previous reports.^27,29,33^ Monomers with asymmetric side-chains were synthesized by purposefully stopping the reaction before full conversion or by lowering the molar equivalents of 1-decanol or triethylene glycol monomethyl ether. The reaction mixture of starting material, monosubstituted, and disubstituted products were separated by column chromatography and/or Kugelrohr distillation. Typically, acid-catalyzed transetherifications involving 3,4-dimethoxythiophene are performed by refluxing in toluene and occasionally purging the reaction with argon to help drive off methanol, such as described for AcDOT-[C_10_]_2_ in the Supporting Information. We found that those standard conditions resulted in a product mixture with more impurities and yields below 15% for AcDOT-[OE_3_]_2_. We hypothesize that methanol was not being sufficiently removed from the solution due to polar interactions with the triethylene glycol monomethyl ether or because the forward and reverse reaction kinetics were comparable for substituting a methoxy for OE_3_ (forward) or substituting an OE_3_ for a methoxy (reverse). This makes the reaction more sensitive to residual methanol compared to the standard alkoxy side-chains commonly found in the literature.^34^ A modified method entailed running the reaction in bulk using triethylene glycol monomethyl ether in a slight excess as the solvent. The mixture was heated and a vacuum was applied over the course of the reaction, resulting in a more efficient removal of methanol without removal of starting reagents or final products. This procedure produced AcDOT-[OE_3_]_2_ monomer in yields exceeding 70% even at scales greater than 5 g. ^1^H and ^13^C nuclear magnetic resonance (NMR) (Figure S1) are consistent with another preparation of AcDOT-[OE_3_]_2_ made through a halogen substitution route.^35^ The NMR spectra are consistent in their peak shifts, integration of nonequivalent hydrogens, and peak multiplicity. Full details for this procedure and other synthesis can be found in the Supporting Information (Figures S1–S10).

After the synthesis and purification of monomers, polymers were prepared by direct (hetero)­arylation polymerization (DArP). Polymers were made from the dihydro- and dibromo monomers, allowing for the control of regio-symmetry and polarity through side-chain substitution, as illustrated in Figure 2. Symmetric side-chain substitution on the thiophene resulted in regio-symmetric polymers (PAcDOT-[C_10_]_2_ and PAcDOT-[C_10_]_2_-*co*-[OE_3_]_2_) while monomers with asymmetric side-chain attachment at the 3- and 4-positions of the thiophene resulted in regio-asymmetric polymers (PAcDOT-[CH_3_-C_10_] and PAcDOT-[CH_3_-C_10_-*co*-CH_3_-OE_3_]). Polymers were designed to better understand how side-chain interactions and regio-symmetry impacted ordering across multiple length scales in the solution and solid-state, as well as the resulting redox properties. For the sake of brevity, the PAcDOT modifier will be dropped and the side-chain abbreviations will be used to identify the polymer structures.

**Figure 2:**
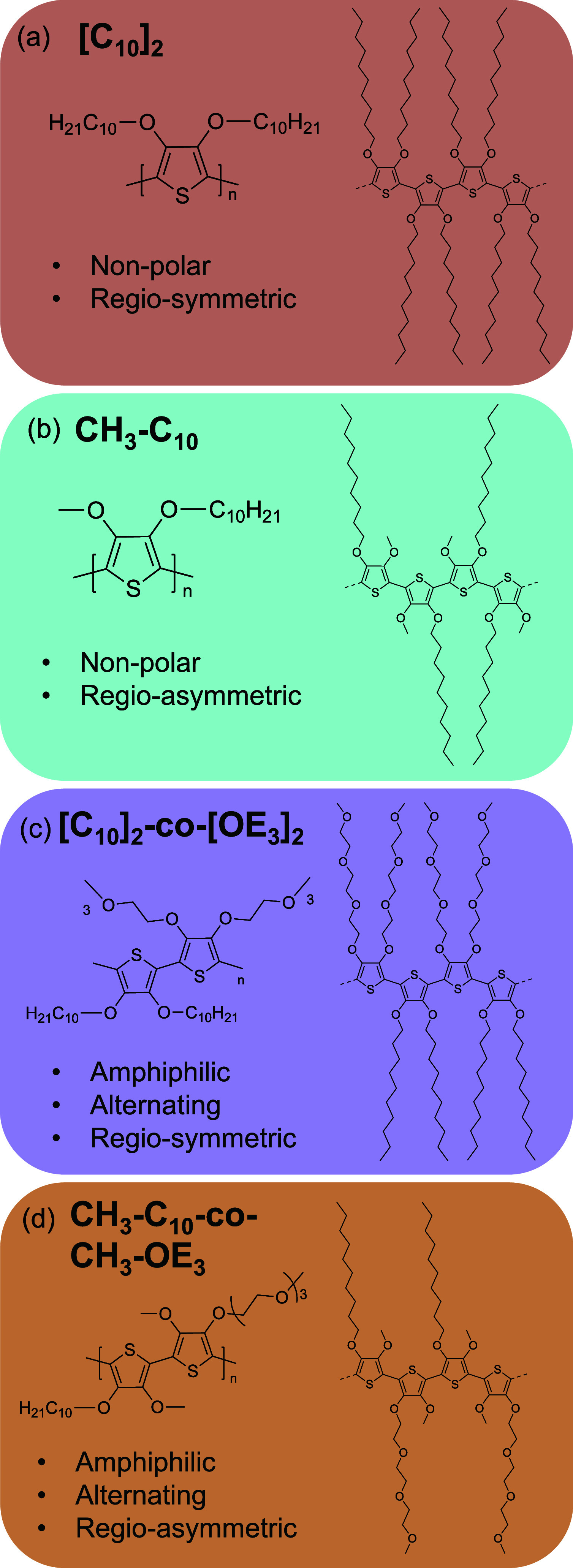
Polymer family investigated in this study showing (a) [C_10_]_2_ (nonpolar, regio-symmetric), (b) [CH_3_-C_10_] (regio-asymmetric, nonpolar), (c) [C_10_]_2_-*co*-[OE_3_]_2_ (regio-symmetric, amphiphilic), and (d) [CH_3_-C_10_-*co*-CH_3_-OE_3_] (regio-asymmetric, amphiphilic) PAcDOTs. Asymmetric side-chain substitution at the 3,4-positions of the thiophene results in regio-asymmetric repeat units. Structures on the right of each repeat unit represent potential segments along the polymer chain. Regio-symmetric structures have one possible side-chain configuration along the backbone. Lack of specificity for addition through the 2 or 5 position in monomers with asymmetric side-chains during polymerization yields more randomness in the side-chain configuration along the backbone.

### Thermal Properties of PAcDOTs

The number-average molecular mass and dispersities (measured by CHCl_3_ gel permeation chromatography (GPC) at 40 °C and referenced to poly­(styrene) standards), degradation temperature (*T*_d_) measured by thermogravimetric analysis (TGA), and peak temperature (*T*_m_) from DSC are shown in Table 1.

**Table 1:** Molar Mass and Thermal Properties of the PAcDOTs

polymer	*M*_n_ (kg/mol)	*Đ*	*T*_d_ (°C)	*T*_m_ (°C)
[C_10_]_2_	14.6	1.79	337	154
[C_10_]_2_-*co*-[OE_3_]_2_	30.0	1.75	337	147
[CH_3_-C_10_]	10.6	1.56	342	219
[CH_3_-C_10_-*co*-CH_3_-OE_3_]	14.0	2.15	322	220

All polymers were soluble in the CHCl_3_ fraction of the Soxhlet extraction and exhibited monomodal GPC chromatograms. We postulate that the combination of a higher number of side-chains per thiophene and the presence of polar oligoether side-chains increased the solubility of [C_10_]_2_-*co*-[OE_3_] during the polymerization, allowing it to reach higher molar masses during the polymerization. At the end of the polymerization, [C_10_]_2_ and [CH_3_-C_10_] resulted in viscous solutions with little precipitated polymer, while both amphiphilic PAcDOTs reached gelation.

Degradation temperatures and thermal transitions were determined by TGA and DSC. All polymers degrade under inert atmosphere between 320 and 340 °C (Figure S11) and have distinct melting and crystallization transitions. After the main thermal degradation, approximately 20% of mass remains for regio-symmetric PAcDOTs and 30% of mass remains for regio-asymmetric PAcDOTs. This corresponds with the complete removal of alkoxy side-chains, leaving the conjugated thiophene core. We note approximately 5% mass loss of the regio-asymmetric [CH_3_–C_10_-*co*-CH_3_-OE_3_] up to 230 °C, which is the annealing temperature. Limited changes are observed to the chromophore absorption after annealing, suggesting that the mass loss is a small amount of side-chain, bound water, or other solvent and not due to changes involving the backbone conjugation. Elevated levels of C and H in elemental analysis potentially indicate residual 1-decanol, which may explain the early mass loss in TGA and bimodal crystallization behavior in DSC. The regio-symmetric polymers have melting temperatures approximately 70 °C lower than the corresponding regio-asymmetric polymers (Figures S12 and S13 and Table S1). All four polymers exhibit significant undercooling for the highest exotherm, consistent with nucleation of a high-temperature crystal form. Similarly, all four polymers exhibit significant entropy of fusion, again consistent with the highest temperature endotherm arising from a crystal melt (Table S2). Unlike other PAcDOTs studied, [C_10_]_2_ has a second transition near 50 °C, attributed to side-chain melting. After side-chain melting, there is a broad melting peak near 130 °C, followed by a sharper melting peak near 155 °C. [C_10_]_2_-*co*-[OE_3_]_2_ has a similar sharp melting and crystallization behavior, without the clear side-chain melt at 50 °C. The broad melting peaks near 130 °C may be due to dispersity or melting–recrystallization–melting processes that can occur at temperatures just below the phase transition.

Temperature-dependent CPOM was performed on regio-symmetric PAcDOT melt-pressed films to corroborate DSC results and aid in identification of phase transitions. Consistent with DSC, it is evident that the crystallization at 133 and 124 °C corresponded to the nucleation and growth of birefringent phases in [C_10_]_2_ and [C_10_]_2_-*co*-[OE_3_]_2_, respectively (Figure S14). Following these initial tests, PAcDOTs were made into solutions and processed into thin films to better understand how polymer structure across multiple length scales evolves during blade coating and thermal treatment.

### Side-Chain-Driven Lyotropic Liquid Crystal Formation

Polymer solutions in CHCl_3_ and thin films blade-coated from CHCl_3_ solutions were further studied using a combination of UV–vis spectroscopy, CPOM, CD, and GIWAXS to better understand differences in how the four polymers assemble in solution and the solid state. Our blade coating parameters (15 mg/mL in CHCl_3_ and 40 mm/s) were in the Landau-Levich (LL) regime due to the fast blade speed in comparison to the solvent evaporation rate. Strained polymer chains under the blade partially relax before solidification due to the blade speed being much faster than evaporation rate.^36^ As a result, this minimizes differences caused by polymer chain alignment during the printing process.

Solution-state UV–vis spectroscopy was performed with a short path cuvette (0.1 mm path length) to allow measurements at concentrations (5 mg/mL) to better match those used during the coating. Comparing the relative intensities of the 0–0 and 0–1 transitions is one indicator of polymer aggregation where a stronger 0–0 absorption generally reflects weaker excitonic coupling and chromophores of longer effective conjugation length.^37,38^ In CHCl_3_, polymers with nonpolar alkoxy side-chains result in a stronger relative 0–0 absorption compared to the amphiphilic counterparts (Figure S15), indicating that the alkoxy side-chains promote interchain interactions that enhance backbone planarity and conjugation.^39–41^ To further probe aggregation, the absorption spectra of lower concentration solutions (0.025 mg/mL in CHCl_3_) were also measured in standard 1 cm cuvettes (Figure S16). The lower concentration suppresses the formation of micron-sized aggregates observed at higher concentrations. Under these dilute conditions, the 0–0 vibronic absorption of [C_10_]_2_ decreases, consistent with a reduced level of aggregation. The vibronic features appear more sensitive to side-chain symmetry, with regio-asymmetric PAcDOTs consistently having a strong 0–0 absorption.

To better mimic the evolution of polymer morphology evolving during printing/coating, solutions at varying concentrations were further analyzed with CPOM and CD to understand how structure changes with increasing concentration as solvent evaporates. Specifically, we are interested in understanding how side-chain chemistry dictates polymer assembly in solution and if the solution morphology persists into the solid state. For example, the presence of ordered solution aggregates, partially attributed to side-chain interactions and noncovalent backbone S–O interactions, can nucleate order in the solid state. Lyotropic phases can also induce order through templating the growth of the solid phase. Indeed, when increasing the concentration of amphiphilic, regio-symmetric [C_10_]_2_-*co*-[OE_3_]_2_ in CHCl_3_, we observe the emergence of birefringence and a nonzero *g*-factor attributed to the formation of chiral, lyotropic LCs in CHCl_3_ (see Figure 3). The *g*-factor is the CD signal normalized by solution/film absorption, which corrects for differences in concentration and thickness.^42^ Moreover, CPOM shows the emergence of nematic tactoids that form from the isotropic solution starting at 50 mg/mL, indicating a transition to a lyotropic LC phase (Figure 3b). Individual tactoids are sites of local order that induce the observed spherical or spindle-shaped birefringent structures. As the concentration steadily increases, the *g*-factor becomes stronger, and the solution remains strongly birefringent. At concentrations above 50 mg/mL, the combination of Schlieren texture under cross-polarizers and a nonzero *g*-factor confirms the formation of a chiral, lyotropic LC mesophase.^43–46^ This chiral emergence in solutions and films of achiral conjugated polymers has been previously reported in the literature and is attributed to the formation of supramolecular structures that adopt helical conformations with a dominant handedness.^45^ Such supramolecular chirality in conjugated polymers relies on the hierarchical assembly of achiral polymer chains into mesoscale chiral structures and is extremely dependent on the processing and structure. As we are not changing the chemical structure of the conjugated chromophore, understanding how side-chains drive supramolecular assembly will allow us to better understand how supramolecular structures influence interactions with light and/or holes/electrons.^44,45,47^

**Figure 3:**
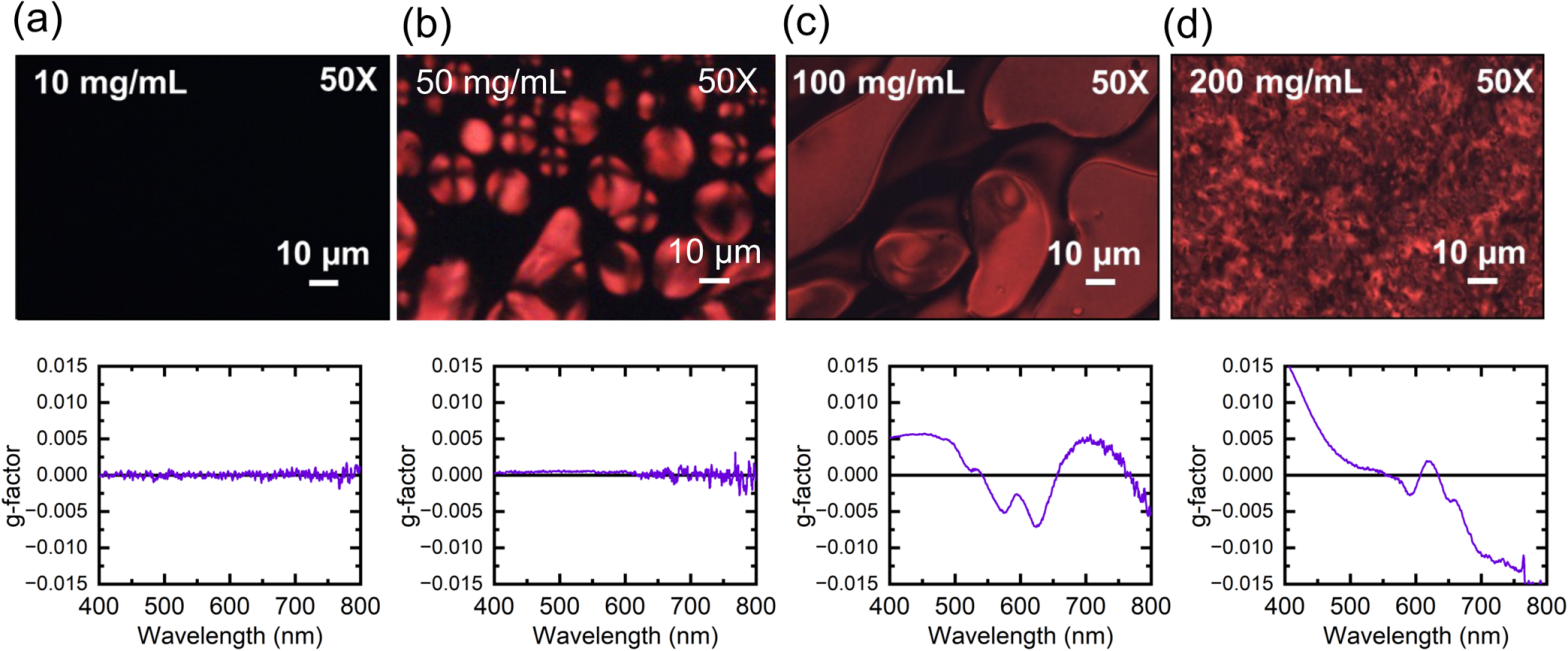
Cross-polarized optical microscopy images of solutions consisting of CHCl_3_ and [C_10_]_2_-*co*-[OE_3_]_2_ and their corresponding *g*-factor at concentrations of (a) 10, (b) 50, (c) 100, and (d) 200 mg/mL. Solutions are isotropic at 10 mg/mL and have no observable CD ellipticity. At 50 mg/mL, tactoids begin to form in solution, although their concentration is not high enough to lead to significant *g*-factor. Further growth and coalescence of the tactoids results in a strong birefringence and *g*-factor, signaling the formation of a lyotropic liquid crystal mesophase with chiral emergence.

Besides [C_10_]_2_-*co*-[OE_3_]_2_, all other PAcDOTs show stronger 0–0 vibronic absorption in solution, indicating weaker excitonic couplings and longer effective conjugation lengths. However, these aggregated solutions do not show solution birefringence, Schlieren-like textures, nor a nonzero *g*-factor, indicating the lack of lyotropic phases and chirality (Figure S17). [C_10_]_2_ is heavily aggregated at room temperature in CHCl_3_, as seen by the micron-sized polymer aggregates in optical microscopy images (Figure S17a). Neither of the regio-asymmetric PAcDOTs evolve birefringent phases when monitoring the meniscus of a drying drop, indicating the lack of LC phases across all concentrations and into the solid-state (Figure S17b,c).^45^ Despite being amphiphilic, the regio-asymmetric [CH_3_-C_10_-*co*-CH_3_-OE_3_] also does not form any chiral mesophases. This result highlights that side-chain amphiphilicity may not be the only driving force to form supramolecular chiral structures and that symmetry also plays a vital role.

### Effects of Thermal Annealing on Film Structure

After studying solution properties, we turn our focus to understanding the polymer film structure in both the as-cast and thermally annealed states. As-cast films were blade-coated in the LL regime and studied without further modifications or post-treatments. Thermal annealing of the films was performed by complete melting followed by slow cooling to room temperature at approximately 3 °C/min. CPOM and CD are used to understand how the solution structure carried over to solid films and, particularly, if the chiral lyotropic LC mesophase of [C_10_]_2_-*co*-[OE_3_]_2_ persists through blade coating and annealing. Figure 4 shows the as-cast CPOM images of thin films as well as the film *g*-factor extracted from CD measurements. Both regio-symmetric PAcDOTs displayed birefringence in the solid state, which points to large crystalline domains that can rotate light through-plane. Regio-asymmetric PAcDOTs have little to no birefringence but are crystalline by DSC (as indicated by the significant enthalpies associated with the cooling exotherms and heating endotherms; this will be discussed in detail later), indicating that the crystallite size is too small for optical detection. As seen in the *g*-factor in Figure 4b, [C_10_]_2_-*co*-[OE_3_]_2_ retains chirality in the solid-state, indicating that the chiral, lyotropic solution structure templates the solid film morphology.

**Figure 4:**
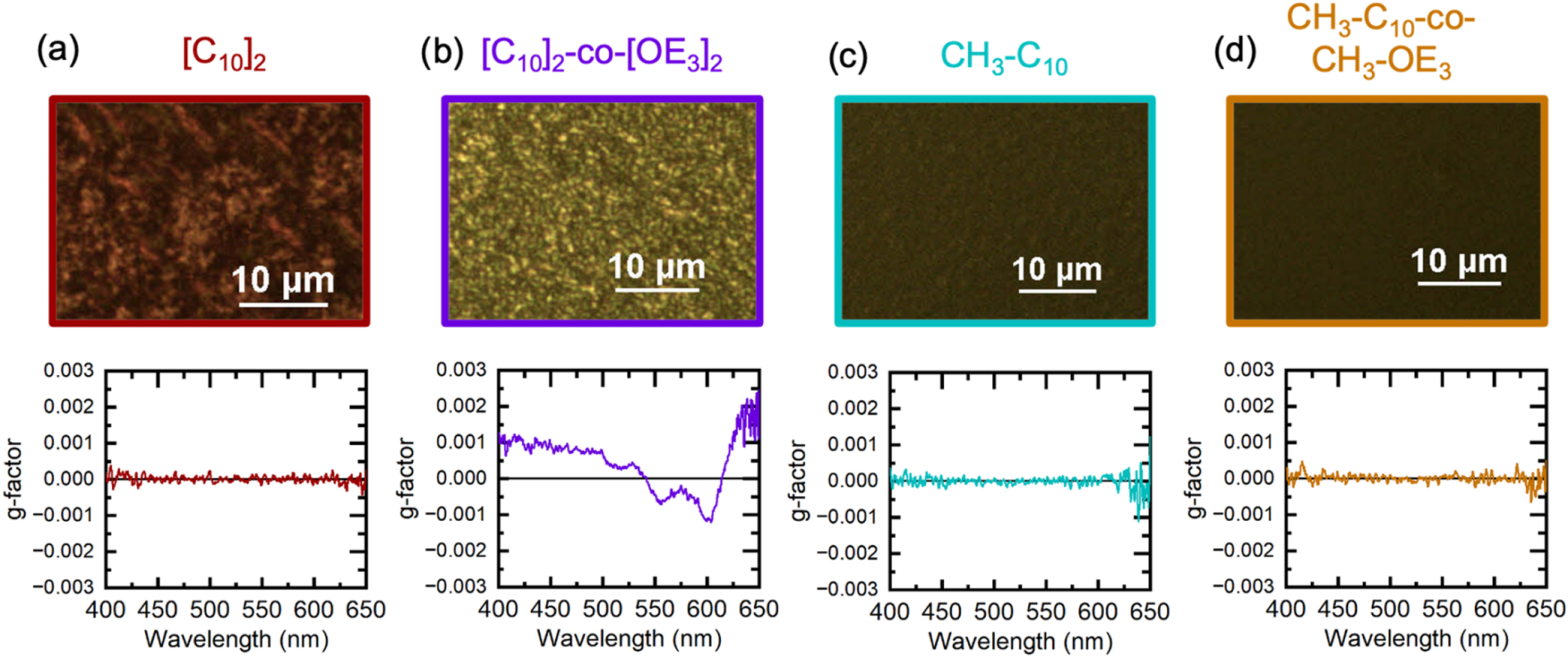
Cross-polarized optical microscopy images and the corresponding *g*-factor for (a) [C_10_]_2_, (b) [C_10_]_2_-*co*-[OE_3_]_2_, (c) [CH_3_-C_10_], and (d) [CH_3_-C_10_-*co*-CH_3_-OE_3_] PAcDOTs. Regio-symmetric side-chain substitution resulted in birefringence, while only the regio-symmetric, amphiphilic [C_10_]_2_-*co*-[OE_3_]_2_ displayed chirality. This chiral structure is thought to carry over from the chiral lyotropic solution mesophase formed during solidification.

Thin film UV–vis–NIR spectroscopy confirmed that all polymer films have similar optical bandgaps with only slight variations in the relative intensities of the 0–0 and 0–1 vibronic peaks (Figure S18). Notably, while [C_10_]_2_-*co*-[OE_3_]_2_ displays weaker relative 0–0 absorptions in solution, indicative of strong excitonic coupling and a shorter relative conjugation length, the corresponding as-cast film has the strongest relative 0–0 absorption of all the PAcDOTs. This points to a large conformational reorganization as the polymer solution transverses to the solid state. Upon annealing, [C_10_]_2_-*co*-[OE_3_]_2_ shows significant changes in its vibronic structure, implying that there is a distinct difference between the as-cast and annealed morphologies. In contrast, the other PAcDOT films showed minimal spectral changes after annealing, suggesting similarity between the as-cast and annealed morphologies (Figure S18).

GIWAXS was performed on all as-cast and annealed PAcDOT films on polished Si substrates to investigate how polymer chains pack in ordered domains. While UV–vis–NIR probes both ordered and disordered chromophores responsible for light absorption, GIWAXS exclusively probes the ordered regions of thin films from the subnanometer to few nanometer length scales. Of particular interest was to probe how the side-chain containing lamellar regions packed based on side-chain symmetry and structure. Figure S19 shows the 2D GIWAXS patterns for as-cast and annealed films at room temperature, and Table S3 shows the (100) out-of-plane lamellar stacking distances. All four polymers exhibit clear diffraction features, consistent with the DSC evidence for crystalline phases. Scattering intensity from the 2D images shows [C_10_]_2_ as mostly isotropic, while [C_10_]_2_-*co*-[OE_3_]_2_ is bimodal, with distinct in-plane and out-of-plane lamellar forms. Regio-symmetric PAcDOTs have an approximately 20–30% larger (100) lamellar stacking distance compared to the regio-asymmetric polymers, which are weakly edge-on in as-cast films. This is attributed to the higher number of side-chains in the regio-symmetric polymers causing steric interactions that promote less tilted side-chains. Side-chain tilt in regio-asymmetric PAcDOTs decreases the lamellar spacing as does incorporation of oligoether side-chains, potentially due to C–O bonds having lower barriers to rotation and more conformational flexibility. It is notable that there is no evidence of lamellar doubling in the polar substituted films (Janus structure). While we cannot exclude the formation of a perfectly centered unit cell with extinction of the odd order reflections, the high similarity of the polar and nonpolar structures suggests no segregation of the OE_3_ layers. Both symmetric PAcDOTs exhibit an in-plane feature near 1.61 Å^–1^ that we attribute to the (002) diffraction of the chain-axis bithiophene repeat unit. The absence of the (001) suggests a pseudo screw symmetry also observed for P3HT. This feature is absent in the asymmetric polymers, suggesting the lack of side-chain symmetry breaks up the *c*-axis diffraction. The [C_10_]_2_ and the two asymmetric polymers have a strong in-plane feature at 1.45 Å^–1^ that is ascribed to the π-π ordering (010). This feature is absent in [C_10_]_2_-*co*-[OE_3_]_2_ due to the presence of complex 3D ordering as discussed below.

The most prominent feature in the as-cast films was the observation of off-axis scattering features in [C_10_]_2_-*co*-[OE_3_]_2_ (Figure 5a). These peaks appear at 1.5 Å^–1^ (4.2 Å in real space) at an azimuthal angle of 40° from the out-of-plane direction. These tilted peaks have been observed in other systems such as bent-core mesogenic molecules. Assemblies of these molecules were chiral despite not having chiral centers.^48^ In the case of the amphiphilic and regio-symmetric PAcDOT, the polymer chain conformation and chain ordering that results in off-axis scattering is not well understood. Thermal annealing of [C_10_]_2_-*co*-[OE_3_]_2_ films changes GIWAXS scattering (see Figure 5b) and is accompanied by a loss in *g*-factor, suggesting that the chiral phase is metastable (see Figure S20). This result highlights how the blade coating process in the LL regime can result in nonequilibrium morphologies that are kinetically trapped in metastable phases, especially for rapidly drying solvents such as CHCl_3_.

**Figure 5:**
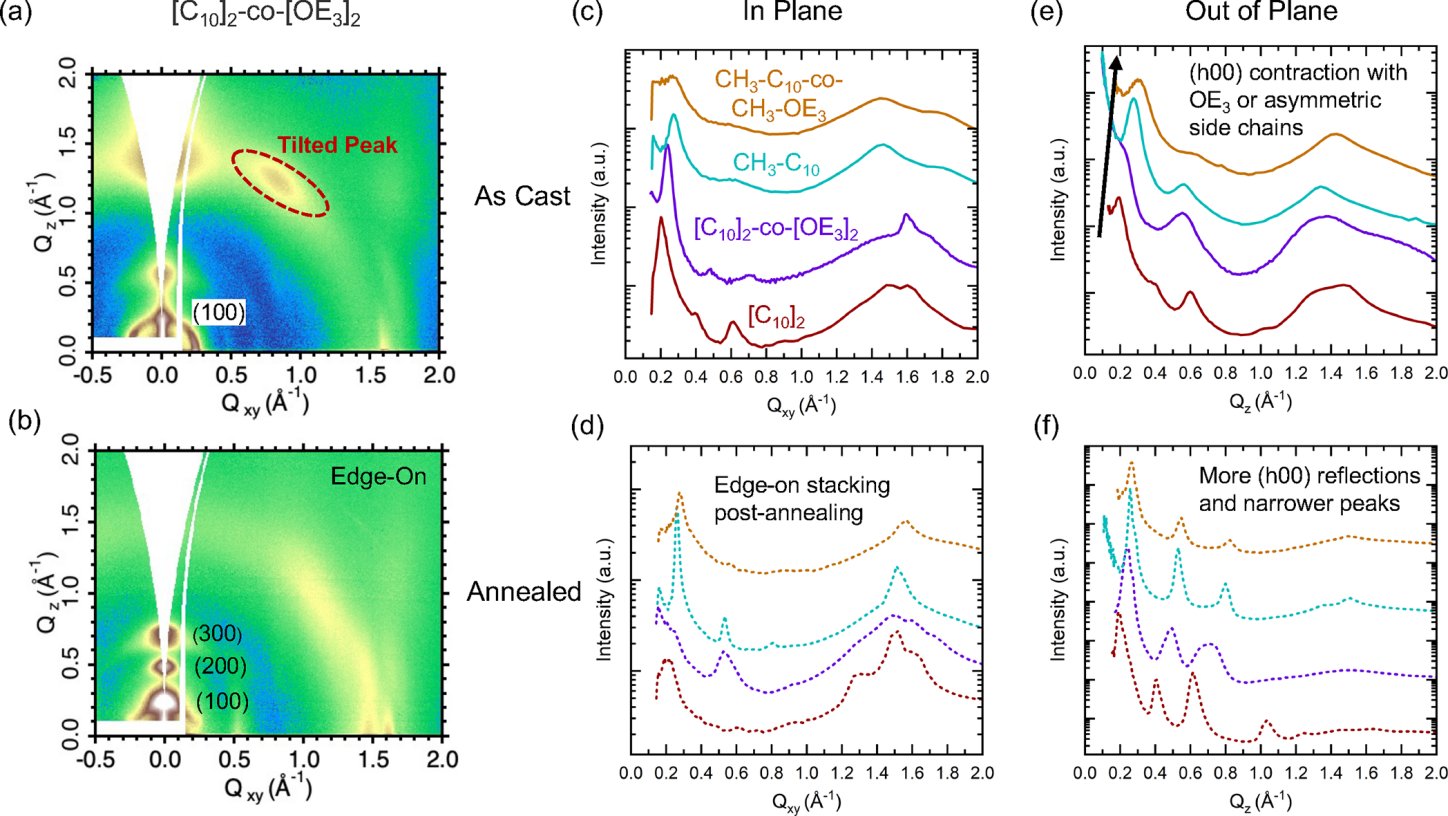
(a) 2D GIWAXS image of as-cast [C_10_]_2_-*co*-[OE_3_]_2_ showing a tilted peak. (b) Annealing the film leads to a highly ordered, edge-on structure coinciding with loss of observed chirality. (c) As-cast films, in-plane (0°), (d) annealed films, in-plane (0°), (e) as-cast films, in-plane (0°) and (f) annealed films, out-of-plane (90°) 1D GIWAXS linecuts. Films become more edge-on after annealing. Amphiphilic or regio-asymmetric side-chains results in shorter lamellar stacking distances. Intensities have been shifted vertically for clarity.

Thermally annealing films by heating to temperatures beyond the backbone melt and allowing chains to order from the melt is a method to eliminate metastable states caused by solution processing. After annealing, all films attained ordered structures as confirmed by the multiple lamellar reflections and narrower peaks in the 1D linecuts of Figure 5c–f. Regio-symmetric [C_10_]_2_ and [C_10_]_2_-*co*-[OE_3_]_2_ became highly edge-on as seen from the transition from widely distributed arcs to compact elliptical diffraction spots along Q_*z*_ (Figure S19). Regio-asymmetric [CH_3_-C_10_] and [CH_3_-C_10_-*co*-CH_3_-OE_3_] were significantly ordered into highly coherent narrow diffraction arcs. The regio-asymmetric PAcDOT films show limited change in birefringence or texture upon annealing, indicating that the crystallites are too small for optical detection (Figure S21).

To further explore the solvent–polymer interactions giving rise to the chiral [C_10_]_2_-*co*-[OE_3_]_2_ film, methanol (MeOH) was introduced at 25 or 50% (polymer concentration was maintained at 15 mg/mL) by volume to the casting solutions. Methanol is more polar and a better solvent for the oligoether side-chains compared to the alkoxy side-chains. At 25 vol % MeOH in CHCl_3_, the *g*-factor became noisier with less clear evidence of chirality (Figure S22). Further increasing the MeOH fraction to 50% by volume eliminates the solution chirality forming achiral microscopic aggregates. This is accompanied by the disappearance of tilted peaks in GIWAXS and the formation of a more dominant amorphous halo (Figure S23). This observation strongly supports that the film morphology in the as-cast state can be regarded as a nonequilibrium metastable phase induced by interactions between CHCl_3_ and the regio-symmetric, amphiphilic side-chains of [C_10_]_2_-*co*-[OE_3_]_2_.

### Nonequilibrium Structures Probed by In Situ GIWAXS

The discovery of the [C_10_]_2_-*co*-[OE_3_]_2_ metastable chiral phase, and large degree of order postannealing, warranted additional investigation. This was done by probing the structural evolution with GIWAXS as the films were heated and cooled to mimic the annealing process. 2D GIWAXS patterns were taken during a heating and cooling cycle at 5 °C/min, allowing for insight into the polymer ordering and phase transitions. Changes in the out-of-plane (100) *d*-spacings and coherence lengths (2π/fwhm, fwhm = diffraction peak full width at half-maximum) at various temperatures during the annealing process provide insights into the side-chain packing and degree of order in crystalline regions. Shorter coherence lengths along the lamellar stacking direction of the crystal lattice represent a more disordered crystal with a higher density of defects or imperfections, whereas the *d*-spacing is determined by how tightly the polymer chains pack.^49,50^ By combining results from variable heating and cooling rate DSC with those obtained during the in situ GIWAXS experiments, we can then interpret phase transitions present during the annealing process/structural evolution.^51,52^

Figure 6 shows 2D GIWAXS images taken at select temperatures during the first heating and cooling cycles. Regio-asymmetric [CH_3_-C_10_] has monotonic thermal expansion during heating, as seen by the (*h*00) reflections going to lower values along the Q_*z*_ direction in temperature-dependent 1D linecuts (Figure S24). The as-cast film does not significantly change until 50 °C, where the film orders and vertical coherence length increases, consistent with thermal expansion and the input of thermal energy allowing crystal thickening and ordering.^22,53^ When cooling from the melt, the structure contracts, and an edge-on structure is formed as evidenced by the high intensity of (h00) reflections along the Q_*z*_ direction and the sharp π–π stacking peak along the Q_*xy*_ direction. During the annealing process, the lamellar vertical coherence length increases dramatically from 8.4 nm in the room temperature as-cast film to 42 nm in the room temperature annealed film. Annealing also results in an expansion of the out-of-plane (100) lamellar peak, increasing from 22.5 Å in the as-cast state to 24.2 Å in the annealed state. This could be attributed to less side-chain tilt in the highly ordered annealed film. For reference, RR-P3HT (*M*_n_ = 26.4 kg/mol and *Đ* = 1.4) was reported to have a lamellar coherence length of 19.2 nm after annealing.^50^ The asymmetrical [CH_3_-C_10_-*co*-CH_3_-OE_3_] has very similar structural evolution, albeit shorter lamellar vertical coherence (11 nm) and lamellar stacking distance (22.4 Å) based on the 2D GIWAXS patterns shown in Figure 6b and 1D GIWAXS linecuts in Figure S25. The high molar entropy, lack of birefringence, and crystallization undercooling point to the regio-asymmetric PAcDOTs having typical semicrystalline phase behavior with small crystals of high local order. This result is quite remarkable as neither polymer has any intentional regio-symmetry along the polymer backbone.

**Figure 6:**
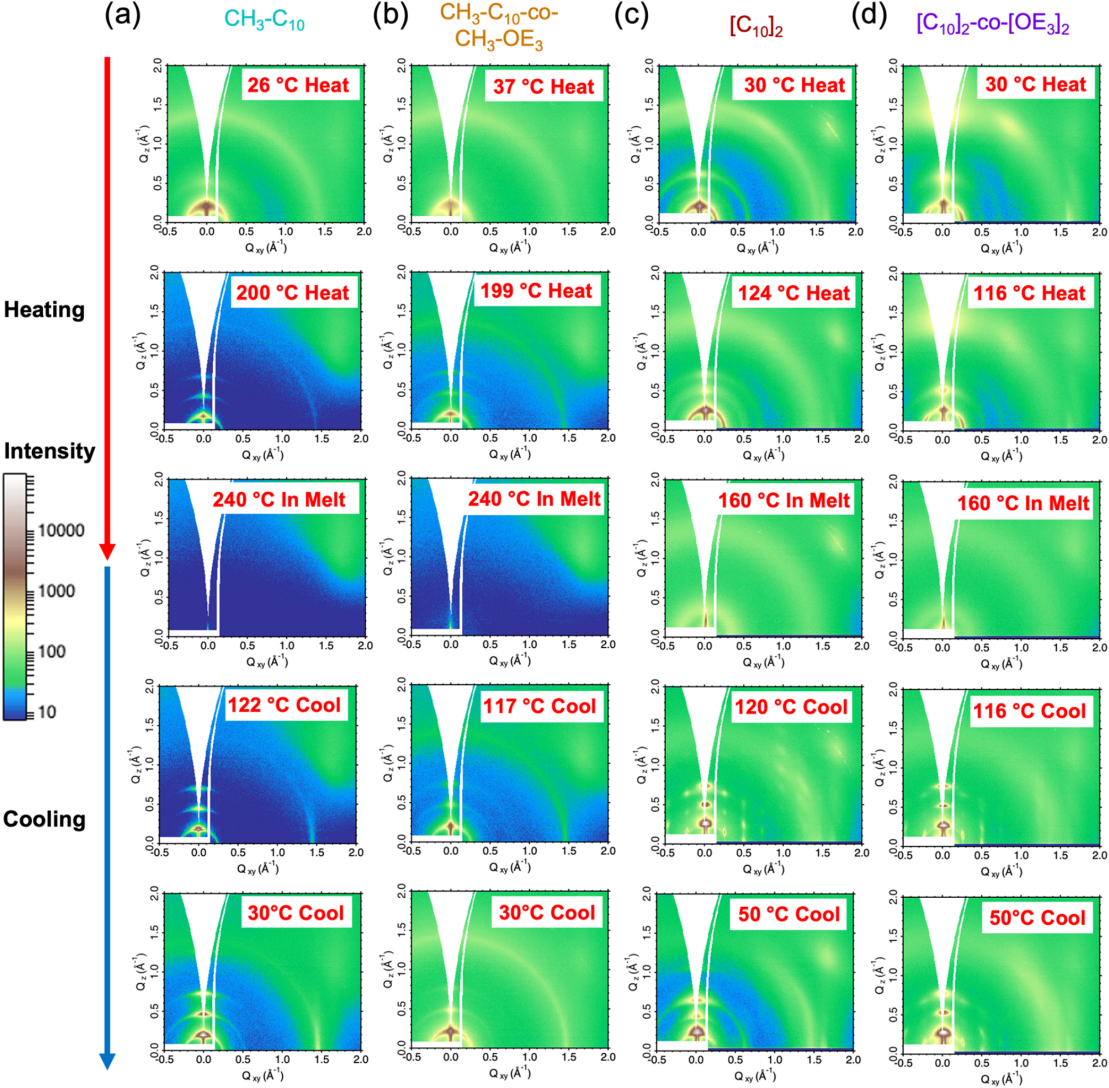
(a) 2D GIXWAS scattering patterns of [CH_3_-C_10_] showing the morphology evolution from the as-cast to the annealed state. Higher lamellar ordering is present in the annealed film, and the lamellae thermally expand upon heating. (b) 2D GIWAXS scattering patterns of [CH_3_-C_10_-*co*-CH_3_-OE_3_], highlighting similar structural morphologies as [CH_3_-C_10_]. (c) 2D GIWAXS scattering patterns of [C_10_]_2_, showing how the as-cast morphology melts, then crystallizes into an intermediate phase with 3D order. (d) 2D GIWAXS scattering patterns of [C_10_]_2_-*co*-[OE_3_]_2_ showing a similar structural evolution as [C_10_]_2_ but maintaining 3D order down to lower temperatures.

In contrast to the regio-asymmetric polymers, [C_10_]_2_ exhibits a richer thermal behavior, as evidenced in Figures 6c and S26. Upon heating to around 100 °C, the out-of-plane lamellar regions contract into a more tightly packed structure. This is most clearly observed by the isosbestic point in the (300) diffraction (Figure S26), which represents a conversion between phases. Cooling from the melt at 160 °C, a highly ordered crystalline phase is formed at ≈150 °C (see Figure 6c, 120 °C cool). This structure is remarkable for a conjugated polymer, exhibiting a rich set of mixed-index peaks indicative of 3D order (e.g., off-axis diffraction spots that are linear combinations of (*h*00) and (0*k*0) diffraction planes). In this highly ordered phase, the vertical lamellar coherence length increases to 45 nm. Another isosbestic point around 85 °C is observed in the (*h*00) out-of-plane reflections upon cooling (Figure S26, bottom right) and is attributed to a near iso-energetic polymorph as there is no apparent signature in DSC. This transition points to minor change in the crystal lattice that results in the loss of 3D order as seen in Figure 6c (50 °C cool). At lower temperatures, there are competing forces for side-chain and backbone ordering, resulting in less ordered packing and a reduction in vertical coherence length to 17 nm, similar to the vertical lamellar coherence length observed in the as-cast film (16 nm). By evaluating the DSC response at variable scan rates (Figure S27), we see that the degree of undercooling and crystallization is rate-dependent, indicative of semicrystalline nucleation and growth. This behavior is observed for all PAcDOTs in this study. The lack of superheating for [C_10_]_2_ is shown in the DSC traces in Figure S28, a feature often observed in flexible, semicrystalline polymers. Polymers that contain rigid mesogens and have thermotropic LC phase transitions, like pBTTT, can have significant superheating.^52^ A large phase transition enthalpy and molar entropy compared to those observed for LC phase transitions, traditional crystallization undercooling, nucleation, and growth of birefringent crystals under CPOM, and lack of superheating points to a “normal” semicrystalline microstructure with large, highly ordered crystals.^51^ This ability to form relatively larger crystals and reach higher relative degrees of crystallization appears to be enabled by the regio-symmetry of the repeat unit.

In the regio-symmetric, amphiphilic [C_10_]_2_-*co*-[OE_3_]_2_, we observe slight differences in structural evolution compared to [C_10_]_2_ (see Figure S29). In contrast to [C_10_]_2_, which exhibits lamellar contraction upon heating, in [C_10_]_2_-*co*-[OE_3_]_2_, there is monotonic thermal expansion upon heating with no clear phase transition until the melt. Upon cooling from the melt, the highly ordered phase with mixed-index peaks develops, as seen in Figure 6d (116 °C cool). No isosbestic point in the out-of-plane (*h*00) reflections is observed with further cooling (Figure S29), indicating that 3D order persists down to lower temperatures compared to [C_10_]_2_. The highly ordered phase in [C_10_]_2_-*co*-[OE_3_]_2_ persists to 50 °C (see Figure 6d, 50 °C cool), likely due to the weaker driving force for side-chain ordering from alternating alkoxy and oligoether side-chains, allowing the backbone to dominate ordering down to lower temperatures. This substantial increase in order also results in an increase of vertical lamellar coherence length from 16 nm in the as-cast film to 45 nm after cooling from the melt to 50 °C. Weaker side-chain interactions near room temperature seem to allow [C_10_]_2_-*co*-[OE_3_]_2_, [CH_3_-C_10_], and [CH_3_-C_10_-*co*-CH_3_-OE_3_] to maintain the higher-level order brought about with slow cooling from the melt. The relative changes in *d*-spacing of the out-of-plane (100) diffraction and vertical coherence are summarized in Table 2, whereas Table S4 compares the phase transition temperatures observed in DSC and GIWAXS. A full breakdown of GIWAXS peak fitting is described in Note S1.

**Table 2:** Summary of In Situ GIWAXS Results for PAcDOTs

	[CH_3_-C_10_]^a^	[CH_3_-C_10_-*co*-CH_3_-OE_3_]^a^	[C_10_]_2_ ^b^	[C_10_]_2_-*co*-[OE_3_]_2_ ^b^
additional phase other than *T*_m_	X	X	85 °C upon cooling	X
change in (100) *d*-spacing postannealing (%)	7.6	9.3	–7.6	–4.2
change in vertical (100) coherence length postannealing (%)	400	62	6.3	280

a Change in (100) properties calculated from as-cast phase at 30 °C and annealed phase at 30 °C postannealing.

b Change in (100) properties calculated from as-cast phase at room temperature and phase observed at 50 °C postannealing.

### Potential-Dependent Optical Transitions and Redox Behavior

By maintaining a consistent conjugated core across these polymers, we can investigate how the side-chain and polymer morphology influence redox behavior in 0.1 mol/L TBAPF_6_ in propylene carbonate (PC). Similar optical bandgaps (Figure S18) among the PAcDOTs indicate that side-chain structure and processing did not significantly change the backbone conformation or extent of effective conjugation, which suggests that any observed differences on the chromophore length-scale are likely attributed to side-chain-induced morphological differences. Films are processed into their as-cast or annealed state, and then the first oxidation and reduction cycle is probed by differential pulse voltammetry (DPV) to understand how microstructure impacts the oxidation potential (Figure S30). Despite thermal annealing having a large impact on the film morphology and coherence lengths for alkyl and π-π stacking, there is little change in the oxidation potential for the first redox cycle. We do note that oxidation potential is a bulk property that is dependent on, e.g., film morphology, degree of swelling, polymer/electrode adhesion, as well as contact resistance. This means there may be opposing forces that result in a net neutral change in oxidation potential with annealing. However, we do observe that incorporation of OE_3_ side-chains lowered the oxidation potential by 290 mV for regio-symmetric [C_10_]_2_-*co*-[OE_3_]_2_ and 340 mV for and regio-asymmetric [CH_3_-C_10_-*co*-CH_3_-OE_3_] compared to their alkylated equivalents. This is consistent with our previous studies of dioxythiophene polymers in organic electrolyte. Along with slightly more electron donation from the OE_3_, the polar nature of the side-chain also enhances swelling in the PC electrolyte, helping facilitate oxidation due to the close proximity of counterions.^54^ The side-chain substitution pattern also affects the oxidation potential. The asymmetric alkoxy side-chain substitution in [CH_3_-C_10_] lowered the oxidation potential by about 100 mV compared to [C_10_]_2,_ potentially due to similar effects observed in polymers with unsubstituted repeat units.^28,54,55^

Potential-dependent absorption spectra help elucidate how side-chain substitution symmetry and polarity impact the neutral-to-doped conversion occurring during electrochemical doping. As seen in Figure 7a, the normalized absorption at λ_max_ for the regio-asymmetric polymers and the amphiphilic [C_10_]_2_-*co*-[OE_3_]_2_ regio-symmetric polymer dope at lower potentials, consistent with lower oxidation potentials determined by DPV. Aside from annealed [C_10_]_2_, all PAcDOT films (as-cast and annealed) show comprehensive bleaching of chromophores along with the disappearance of vibronic peaks during doping. It has been shown that the most ordered film domains are preferentially doped based on stabilized polaron energy (see Figure S31).^56–59^ However, annealed [C_10_]_2_ does not fully bleach and retains vibronic features even at high potentials, implying that the highly ordered regions of the polymer are not fully doped. [C_10_]_2_ has the largest phase transition enthalpy, which may point to the annealed film having a relatively high degree of crystallinity with ordered side-chain regions. This suggests that while ordering is beneficial for charge carrier stabilization, excessive crystallinity and side-chain density can inhibit the incorporation of electrolyte necessary for complete electrochemical doping for all film regions.

**Figure 7:**
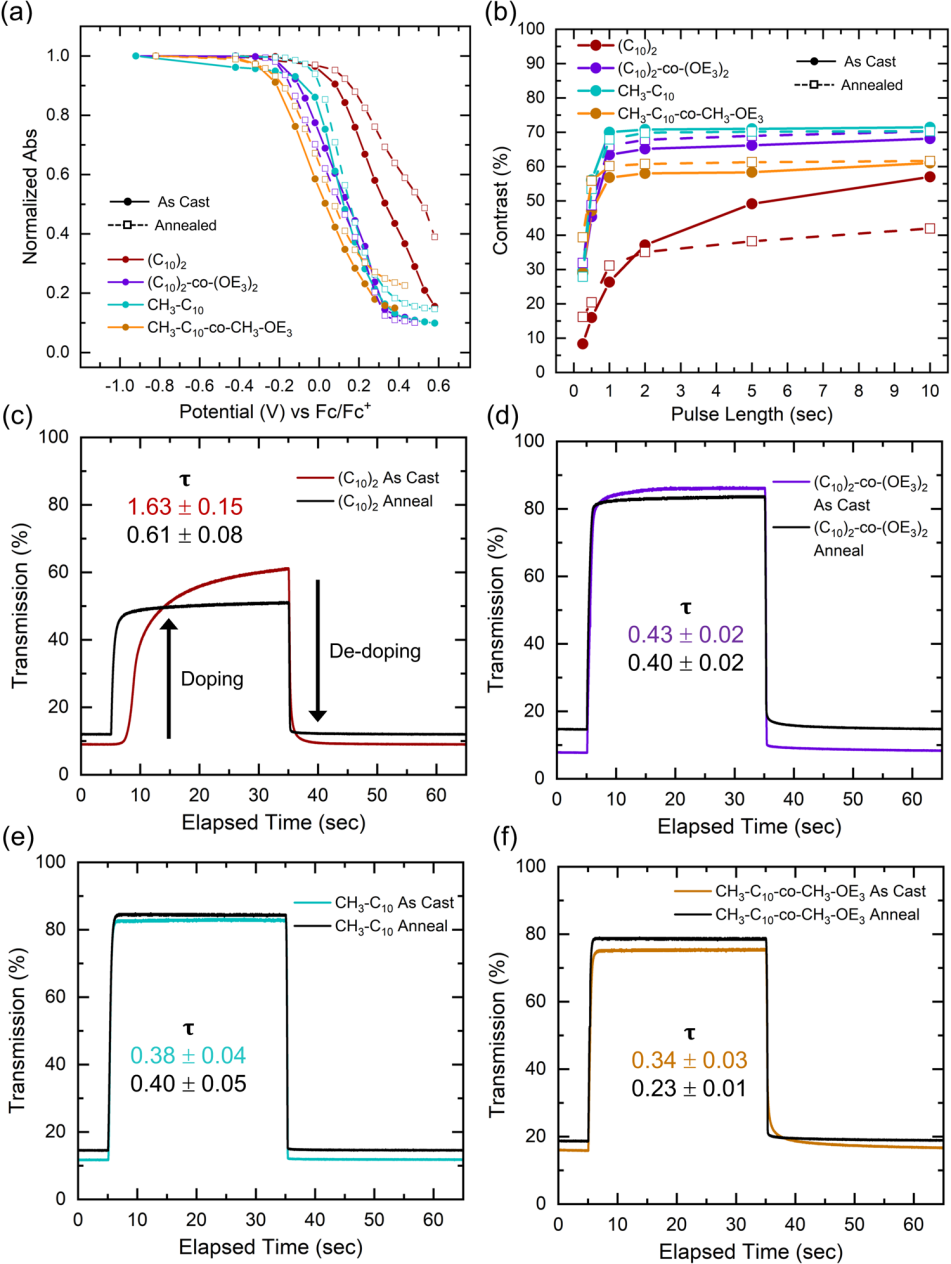
(a) Normalized absorption at λ_max_ as a function of potential. All polymers except annealed [C_10_]_2_ result in highly transmissive states at high potentials. (b) Optical contrast (%*T*_bleach_ – %*T*_color_) achieved after a given oxidation and reduction pulse length. Regio-asymmetric or amphiphilic PAcDOTs maintain higher contrast down to faster switching speeds, indicating improved doping and dedoping kinetics responsible for the switching of chromophores between the bleached and colored state. Optical transmission after a 30 s oxidizing pulse (bleaching) and a subsequent 30 s reducing pulse (coloration) for (c) [C_10_]_2_, (d) [C_10_]_2_-*co*-[OE_3_]_2_, (e) [CH_3_-C_10_], and (f) [CH_3_-C_10_-*co*-CH_3_-OE_3_] PAcDOTs. The time constants for optical switching rates are included for each material.

To probe the electrochemical doping kinetics, here termed the time required to fully bleach the π-π* transition, we applied a series of oxidizing and reducing pulses for varying lengths of time to PAcDOT films. Figure 7b (and Figure S32) shows how amphiphilic side-chains or regio-asymmetric PAcDOTs maintain a larger optical change as a function of pulse length, indicative of a more efficient and complete doping process. This data can be fit to an exponential to calculate a time constant, τ, for the doping kinetics.^60,61^ Time constants for as-cast and annealed [C_10_]_2_ range from 1.6 to 0.6 s, while the regio-asymmetric and amphiphilic polymers exhibited notably faster doping kinetics (τ = 0.23–0.40 s). Fitted plots are shown in Figure S33, and τ for each PAcDOT film is in Table S5. Photographs illustrating film color in the neutral and oxidized states are shown in Figure S34.

Potential asymmetries in the rate of doping and dedoping are readily seen when comparing the change in transmittance at longer pulse lengths (assuming comparable film thicknesses). Figure 7c–f shows the transmission across full oxidation and reduction cycles, each held for 30 s. Amphiphilic or regio-asymmetric PAcDOTs switch the fastest and have the highest optical contrast between the neutral and fully doped states. Annealing [CH_3_-C_10_-*co*-CH_3_-OE_3_] results in an improvement in doping kinetics, which are maintained after electrochemical break-in. This is significant because it indicates that benefits of annealing, such as improved charge transport pathways, are retained after many electrochemical cycles. Another important thing to note is the lack of correlation between the oxidation potential and doping kinetics. [CH_3_-C_10_] dopes at the same rate as [C_10_]_2_-*co*-[OE_3_]_2_ despite having a 200 mV higher oxidation potential. Upon annealing, [C_10_]_2_ exhibits an improvement in the doping kinetics at the expense of complete bleaching of the neutral state. We attribute this to the lack of electrochemical doping of highly ordered regions, observed in spectroelectrochemistry. Enhancement of doping kinetics can also be illustrated by the dependence of peak anodic current on the CV scan rate (see Figure S35). Again, regio-asymmetric or amphiphilic PAcDOTs maintain linearity out to faster scan rates, signifying improved charge transport shown in Figure S36. Because mass transport tends to be orders of magnitude slower than electronic transport, we assume that the differences observed in redox kinetics are likely due to differences in ion migration rates.

## Conclusions and Perspectives

A family of acyclic dioxythiophene polymers were synthesized with either nonpolar or amphiphilic side-chains. Polymers are made either regio-symmetric or regio-asymmetric by controlling the side-chain substitution symmetry during monomer synthesis. The combination of side-chain polarity and repeat unit symmetry influences polymer conformation in CHCl_3_, resulting in a lyotropic LC with chiral emergence for regio-symmetric, amphiphilic [C_10_]_2_-*co*-[OE_3_]_2_ solutions. The chiral structure for [C_10_]_2_-*co*-[OE_3_]_2_ persists from solution to the solid-state after blade coating in the Landau-Levich regime. All other PAcDOTs are achiral in solution and in thin films. Melting and slowly cooling regio-symmetric PAcDOTs results in backbone-driven ordering of a morphology with 3D order. Strong side-chain interactions in [C_10_]_2_ impede backbone order at lower temperatures. Regio-asymmetric PAcDOT films have order and crystallinity, evidenced by large coherence lengths and single backbone melting and crystallization in DSC and GIWAXS. Annealing [CH_3_-C_10_-*co*-CH_3_-OE_3_] films into a more ordered state improves charge transport pathways that are maintained even after electrochemical break-in. However, the impact of side-chain symmetry and polarity is also significant, where the introduction of amphiphilic or regio-asymmetric side-chains results in lower oxidation potentials and a more efficient doping and dedoping process.

Incorporating these design principles of regio-symmetry and amphiphilic side-chains into existing higher-performance structural motifs, such as those with more planar backbones or less side-chain density, may result in polymers with unique solution and solid-state morphologies. Controlling the assembly of conjugated polymers from solution to solid-state, such as creating mesoscale chiral structures from achiral repeat units, is an exciting experimental space that remains largely unexplored and could yield new materials with unique optoelectronic properties.

## Supplementary Material



## Notes

Additional details on materials, synthesis, procedures, characterization, and instrumentation (PDF)
