# Supplementary material for: Impact of Side-Chain Polarity and Symmetry on the Structure and Properties of Acyclic Dioxythiophene Polymers
Source: Chem Mater. 2025 Jun 20;37(13):4593–606. doi: 10.1021/acs.chemmater.4c03394 (PMC12247928; doi:10.1021/acs.chemmater.4c03394)
Supplement: Supplementary file 1 [file cm4c03394_si_001.pdf]

Supplemental Information

Impact of Side-Chain Polarity and Symmetry on the Structure and Properties of Acyclic Dioxythiophene Polymers

*Joshua M. Rinehart[1], Zhuang Xu[2], Ziming Wang[2], Anna M. Österholm[3], Lucas Q. Flagg[4], Lee J. Richter[4], Chad R. Snyder[4], Ying Diao[2,5], and John R. Reynolds[1,3]**

[1]School of Materials Science and Engineering, Georgia Institute of Technology, Atlanta, Georgia 30332, USA, [2]Department of Chemistry, Beckman Institute for Advanced Science and Technology, University of Illinois Urbana–Champaign, Urbana, Illinois 61801, USA, [3]School of Chemistry and Biochemistry, Georgia Institute of Technology, Atlanta, Georgia 30332, USA, [4]National Institute of Standards and Technology, Gaithersburg, Maryland 20899, USA, [5]Department of Chemical and Biomolecular Engineering, University of Illinois Urbana–Champaign, Urbana, Illinois 61801, USA.

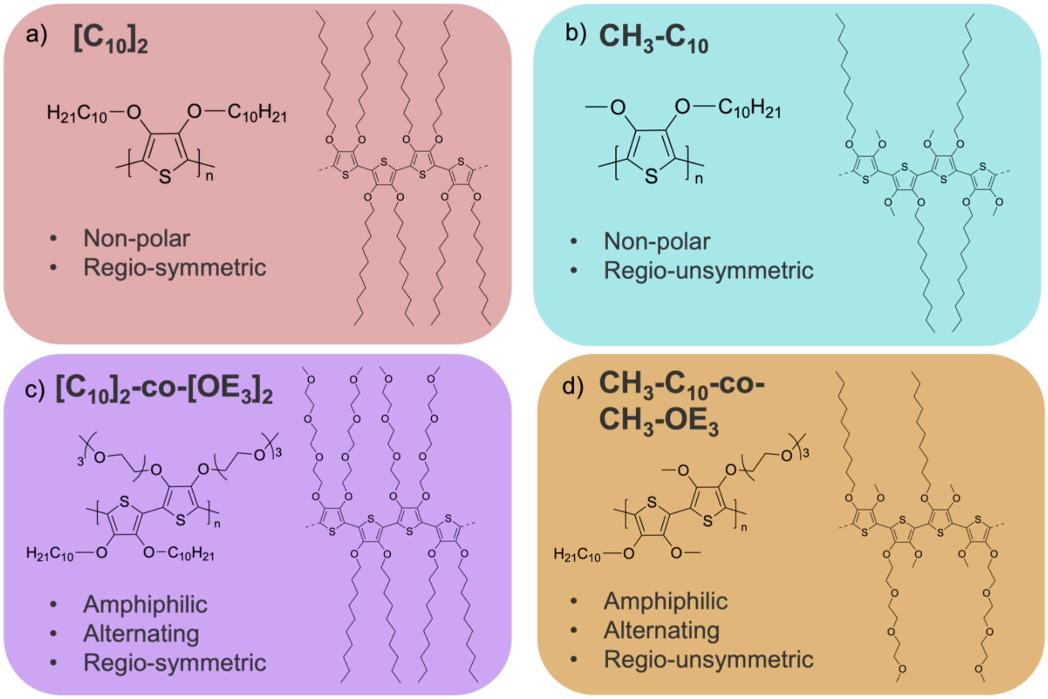

Poly(acyclic dioxythiophene) (PAcDOT) structures and acronyms used in this study.

Materials

Chemicals

All reagents were purchased from commercial sources and used as received unless otherwise stated. Toluene was purified with a MBraun solvent purification system. Anhydrous dimethylacetamide was used for polymerizations. 3,4-dimethoxythiophene and triethylene glycol monomethyl ether were distilled before use.

Monomer Synthesis

AcDOT-[OE_3]$_2$ - Vacuum Assisted Transetherifiction

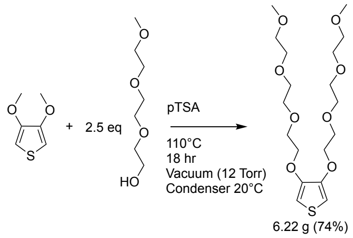

3,4-dimetheoxythiopehene (DMT, 3 g, 20.8 mmol, 1 eq), triethylene glycol monomethyl ether (OE_3-OH, 8.54 g, 52 mmol, 2.5 eq), and para-toluenesulfonic acid monohydrate (pTSA, 0.396 g, 2.1 mmol, 0.1 eq) were added to a 25 mL round bottom flask with magnetic stir bar. A condenser was attached (water set to 20 °C) and vacuum line (connected to Schlenk line) was then attached to top of the condenser, which was set to 12 torr (1600 Pa). The flask was cycled between vacuum and argon 3 times to remove any dissolved gases. Then, the flask was heated at 110 °C for 18 h while under 12 torr of vacuum. After cooling, the reaction was diluted with ether and washed with saturated $NaHCO_3$. The organic layer was extracted 3 times with ethyl acetate. Organic phases were combined, washed with brine, then dried with $MgSO_4$ and filtered. Solvent was removed with rotary evaporation and the resulting viscous oil that was then loaded onto a silica column with pure ether. The column was run in pure ether, resulting in DMT as the first spot and CH_3-OE_3 AcDOT as the second spot. Once AcDOT-[CH_3-OE_3] eluted from column, acetone was added to the column slowly up to 10 % by volume. Rotary evaporation and drying under vacuum at 70 °C overnight yielded AcDOT-[OE_3]$_2$ as pale yellow oil (6.22 g, 74 %). ^{1}H NMR (400 MHz, $CDCl_3$, 25 °C) δ (ppm) 6.26 (2H, s), 4.17 (4H, t, J = 5.2 Hz), 3.87 (4H, t, J = 5.3 Hz), 3.77-3.65 (12H, m), 3.57 (4H, m), 3.40 (6H, s). ^{13}C NMR (100 MHz, $CDCl_3$, 25 °C) δ (ppm) 147.1, 97.8, 71.9, 70.7, 70.6, 70.5, 69.8, 69.5, 59.0. HR-MS (ESI) m/z calcd for $C_{18}H_{32}O_8S$ (M+H): 409.18907, found 409.18908.

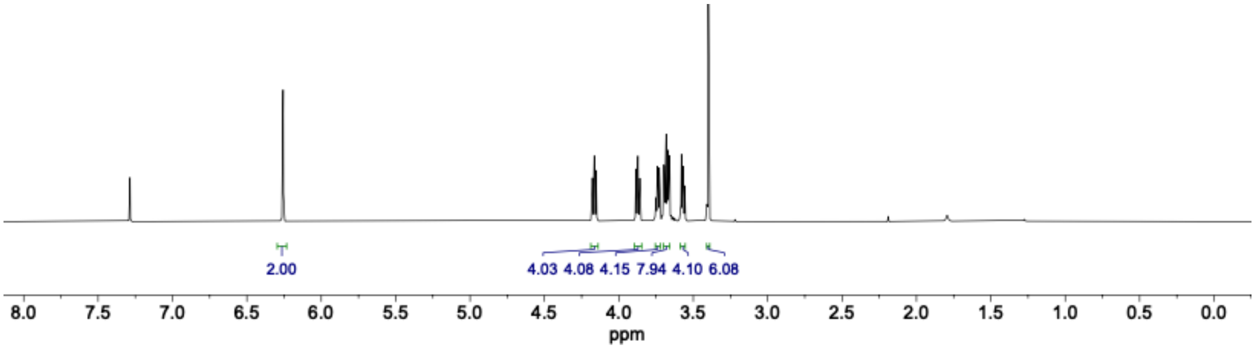

Figure S1. ^{1}H-NMR (400 MHz) spectrum of AcDOT-$[OE_3]_2$ in $CDCl_3$.

<u>AcDOT-[CH_3-OE_3]</u>

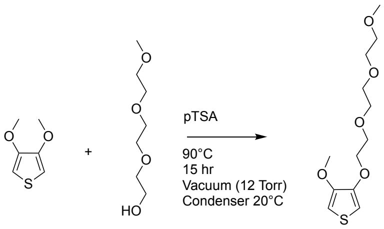

3,4-dimetheoxythiopehene (DMT, 3 g, 20.8 mmol, 1 eq), triethylene glycol monomethyl ether (OE_3-OH, 3.41 g, 20.8 mmol, 1 eq), and para-toluenesulfonic acid monohydrate (pTSA, 0.198 g, 1.05 mmol, 0.05 eq) were added to a 25 mL round bottom flask with magnetic stir bar. A condenser was attached (water set to 20 °C) and vacuum line (connected to Schlenk line) was then attached to top of the condenser, set to 12 torr (1600 Pa). The flask was cycled between vacuum and argon 3 times, then reacted at 90 °C for 15 h while under vacuum. Lower temperatures and fewer equivalents of the triethylene glycol monomethyl ether were used to isolate the mono-substituted product. After cooling, the reaction was diluted with ether and washed with saturated $NaHCO_3$. The organic layer was extracted 3 times with ethyl acetate. Organic phases were combined, washed with brine, then dried with $MgSO_4$ and filtered. Solvent was removed with rotary evaporation and the resulting viscous oil was loaded onto a silica column with pure ether. The column was run in pure ether, resulting in DMT as the first spot and AcDOT-[CH_3-OE_3] as the second spot. Rotary

evaporation and drying under vacuum at 70 °C overnight yielded AcDOT-[CH_3-OE_3] as clear oil (2.0 g, 35 %). ^{1}H NMR (400 MHz, $CDCl_3$, 25 °C) δ (ppm) 6.27 (1H, d, J = 3.3 Hz), 6.20 (1H, d, J = 3.3 Hz), 4.18 (2H, t, J = 5.2 Hz), 3.88 (2H, t, J = 5.3 Hz), 3.86 (3H, s), 3.79-3.64 (6H, m), 3.57 (2H, m), 3.39 (3H, s). ^{13}C NMR (100 MHz, $CDCl_3$, 25 °C) δ (ppm) 148.1, 146.7, 97.6, 96.2, 71.9, 70.8, 70.6, 70.5, 69.8, 69.4. HR-MS (ESI) m/z calcd for $C_{12}H_{20}O_5S$ (M+H): 277.1104, found 277.1102.

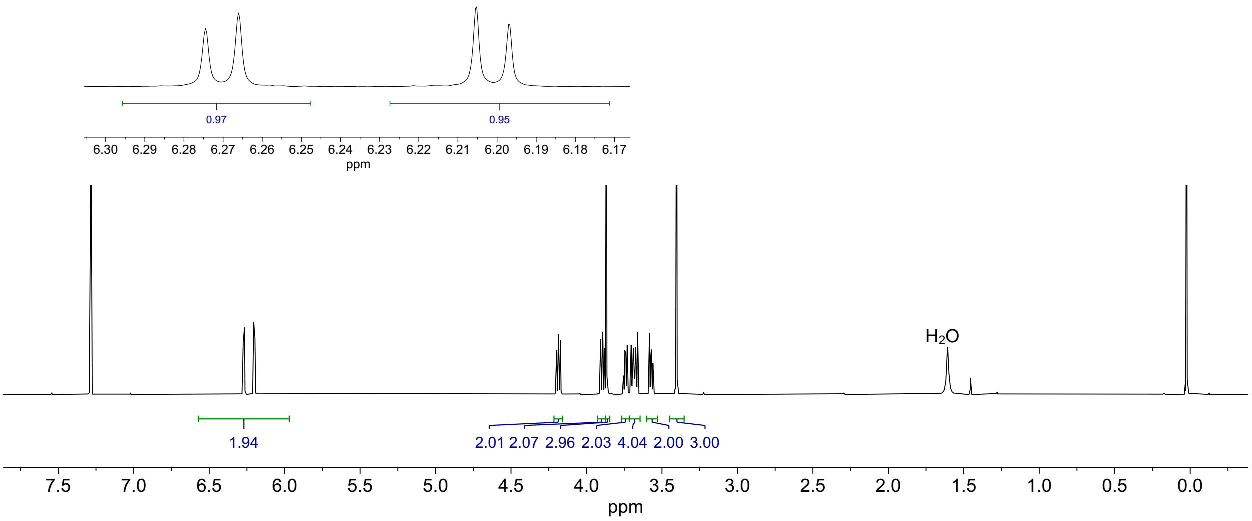

Figure S2. ^{1}H-NMR (400 MHz) spectrum of AcDOT-[CH_3-OE_3] in $CDCl_3$.

<u>AcDOT-[C_{10}]$_2$</u>

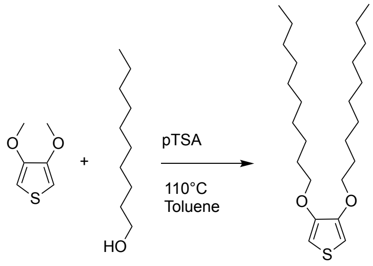

DMT (4 g, 28 mmol, 1 eq), 1-decanol (12.9 g, 81.6 mmol, 2.9 eq), pTSA (0.53 g, 2.8 mmol, 0.1 eq), and toluene (115 mL) were added to a two-neck 250 mL round bottom flask with magnetic stir bar and condenser (septa on the condenser top and remaining flask neck). The flask was purged

with argon for at least 15 min to remove air. The flask was completely sealed from air then immersed in an oil bath to reflux. After a few hours, an argon line was inserted into the condenser septum and vent needle was inserted into the round bottom neck to help drive off methanol and push the equilibrium further towards product for approximately 15 minutes. Any longer time or continuous argon purging resulted in excessive loss of solvent. The reaction was left to reflux overnight, then vented once more (15 minutes) in the morning. The reaction was removed from heat after approximately 18 hours. After cooling, the reaction was diluted with ether and washed with saturated $NaHCO_3$. The organic layer was extracted 3 times with ether. Organic phases were combined, washed with brine, then dried with $MgSO_4$ and filtered. Solvent was removed with rotary evaporation and the resulting viscous oil was loaded onto a silica column with pure hexanes. The column was run in pure hexanes. AcDOT-$[C_{10}]_2$ is collected as the first fraction, then dried to a clear oil which solidified to a white solid in the freezer (5.4 g, 48 %). ^{1}H NMR (400 MHz, $CDCl_3$, 25 °C) δ (ppm) 6.18 (2H, s), 3.99 (4H, t, J = 6.8 Hz), 1.83 (4H, p, J = 6.9 Hz), 1.45 (4H, p, J = 7.0 Hz), 1.29 (24H, b), 0.90 (6H, t, J = 7.0 Hz). ^{13}C NMR (100 MHz, $CDCl_3$, 25 °C) δ (ppm) 147.6, 96.8, 70.6, 32.0, 29.60, 29.58, 29.42, 29.35, 29.05, 26.0, 22.7, 14.1. HR-MS (ESI) m/z calcd for $C_{24}H_{44}O_2S$ (M+H): 397.3135,

found 397.3135.

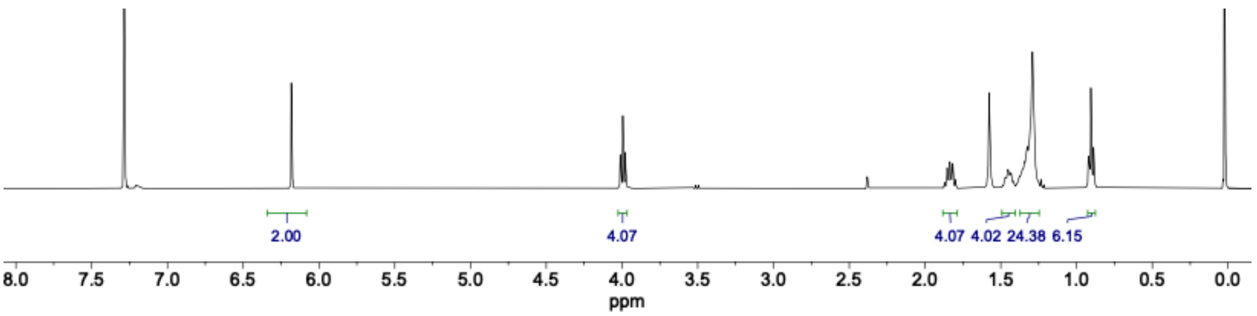

Figure S3. ^{1}H-NMR (400 MHz) spectrum of AcDOT-$[C_{10}]_2$ in $CDCl_3$.

AcDOT-$[CH_3$-$C_{10}]$

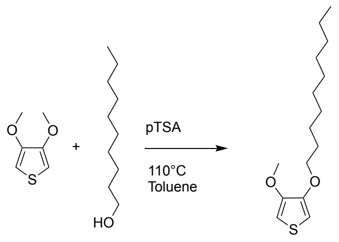

DMT (3 g, 20.8 mmol, 1 eq), 1-decanol (3.29 g, 20.8 mmol, 1 eq), pTSA (0.198 g, 1.05 mmol, 0.05 eq), and toluene (80 mL) were added to a two-neck 250 mL round bottom flask with condenser and magnetic stir bar. The flask was purged with argon for at least 15 min, then the vessel sealed and reacted at 90 °C overnight. A lower temperature was used, and no argon purging was performed so that the mono-substituted product was favored. After cooling, the reaction was diluted with ethyl acetate and washed with saturated $NaHCO_3$ and DI water. The organic layer was extracted 3 times with ethyl acetate. Organic phases were combined, washed with brine, then dried with $MgSO_4$ and filtered. Solvent was removed with rotary evaporation and the resulting viscous oil was loaded onto a silica column with pure hexanes. The column was run in pure hexanes, slowly adding 5 % by volume ethyl acetate. AcDOT-$[CH_3$-$C_{10}]$ is collected as the second fraction by thin layer chromatography. Kugelrohr distillation (130 °C, 2000 Pa) resulted in a clear oil which solidified to a white solid in the freezer (2.3 g, 41 %). ^{1}H NMR (400 MHz, $CDCl_3$, 25 °C) δ (ppm) 6.21 (1H, d, J = 3.2 Hz), 6.19 (1H, d, J = 3.2 Hz), 4.00 (2H, t, J = 6.8 Hz), 3.88 (3H, s), 1.85 (2H,

m), 1.45 (2H, m), 1.29 (12H, b), 0.90 (3H, t, J = 6.8 Hz). ^{13}C NMR (100 MHz, $CDCl_3$, 25 °C) δ (ppm) 148.2, 147.2, 96.8, 96.0, 70.6, 57.5, 31.9, 29.6, 29.55, 29.4, 29.3, 29.1, 26.0, 22.7, 14.1. HR-MS (ESI) m/z calcd for $C_{24}H_{44}O_2S$ (M+H): 271.1726, found 271.1725.

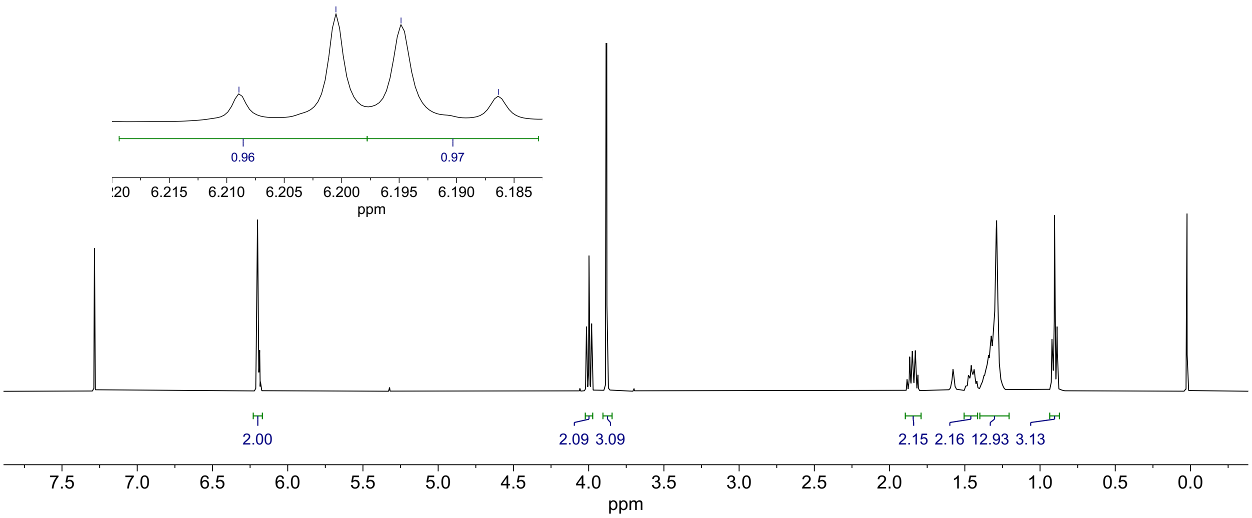

Figure S4. ^{1}H-NMR (400 MHz) spectrum of AcDOT-[CH_3-C_{10}] in $CDCl_3$.

<u>AcDOT-[C_{10}]$_2$-Br_2</u>

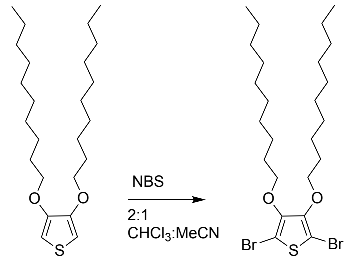

AcDOT-[C_{10}]$_2$ (1.2 g, 3 mmol, 1 eq) was added to a 100 mL round bottom flask with $CHCl_3$ (40 mL). N-bromosuccinimide (NBS, 1.16 g, 6.5 mmol, 2.15 eq) was added to acetonitrile (ACN, 20 mL) and added to a flask and stirred until dissolved. The $CHCl_3$ solution was covered in foil and placed in a salt ice bath at -8 °C. The NBS solution was added, and solution purged with argon. The reaction was stirred and allowed to warm to room temperature overnight. The reaction was diluted with ethyl acetate and washed with saturated $NaHCO_3$ and DI water. The organic layer was

extracted 3 times with ethyl acetate. Organic phases were combined, washed with brine, then dried with $MgSO_4$ and filtered. Solvent was removed with rotary evaporation and the resulting viscous oil was loaded onto a short silica column with pure hexanes. AcDOT-[C_{10}]$_2$-Br_2 was collected as the first spot, dried to a clear oil which solidified to a white solid in the freezer (1.54 g, 93 %). ^{1}H NMR (400 MHz, $CDCl_3$, 25 °C) δ (ppm) 4.08 (4H, t, J = 6.6 Hz), 1.75 (4H, p, J = 6.7 Hz), 1.47 (4H, p, J = 7.8 Hz), 1.30 (24H, b), 0.91 (6H, t, J = 6.9 Hz). ^{13}C NMR (100 MHz, $CDCl_3$, 25 °C) δ (ppm) 147.6, 95.2, 73.9, 32.0, 30.0, 29.65, 29.62, 29.42, 29.38, 25.9, 22.73, 14.1.

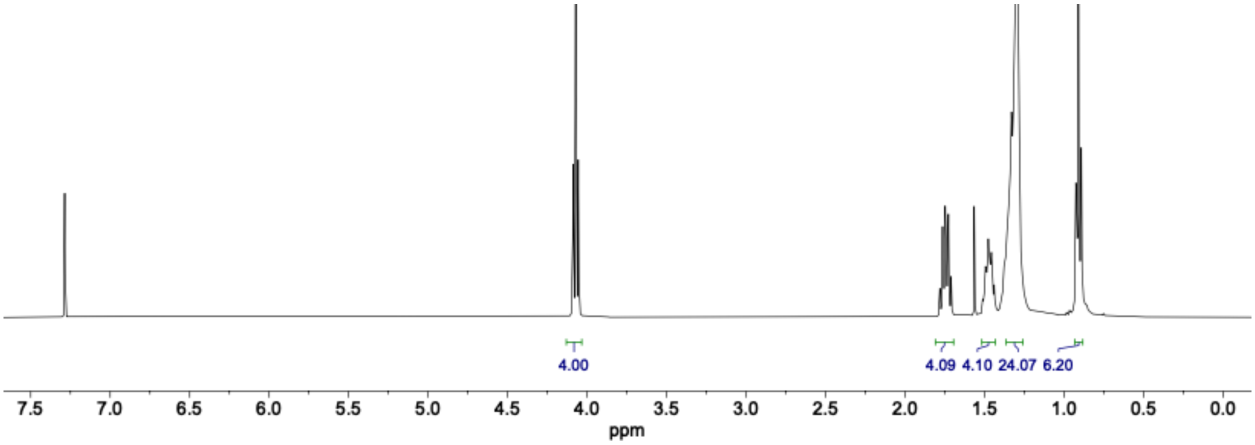

Figure S5. ^{1}H-NMR (400 MHz) spectrum of [C_{10}]$_2$ AcDOT-Br_2 in $CDCl_3$.

<u>AcDOT-[CH_3-C_{10}]-Br_2</u>

AcDOT-[CH_3-C_{10}] (0.6 g, 2.2 mmol, 1 eq) was added to a 100 mL round bottom flask with $CHCl_3$ (20 mL). N-bromosuccinimide (NBS, 0.849 g, 4.7 mmol, 2.15 eq) was added to acetonitrile (ACN, 10 mL) and added to a flask and stirred until dissolved. The same procedure as AcDOT-[C_{10}]$_2$-Br_2 was followed, resulting in a clear oil (0.932 g, 98.9 %). ^{1}H NMR (400 MHz, $CDCl_3$, 25 °C) δ (ppm) 4.08 (2H, t, J = 6.6 Hz), 3.92 (3H, s), 1.76 (2H, p, J = 6.7 Hz), 1.48 (2H, p, J = 7.1 Hz), 1.29 (12H, b), 0.91 (3H, t, J = 6.9 Hz). ^{13}C NMR (100 MHz, $CDCl_3$, 25 °C) δ (ppm) 148.3, 147.4, 95.3, 94.8, 74.0, 60.9, 31.9, 29.96, 29.62, 29.60, 29.37, 29.35, 25.86, 22.73, 14.15.

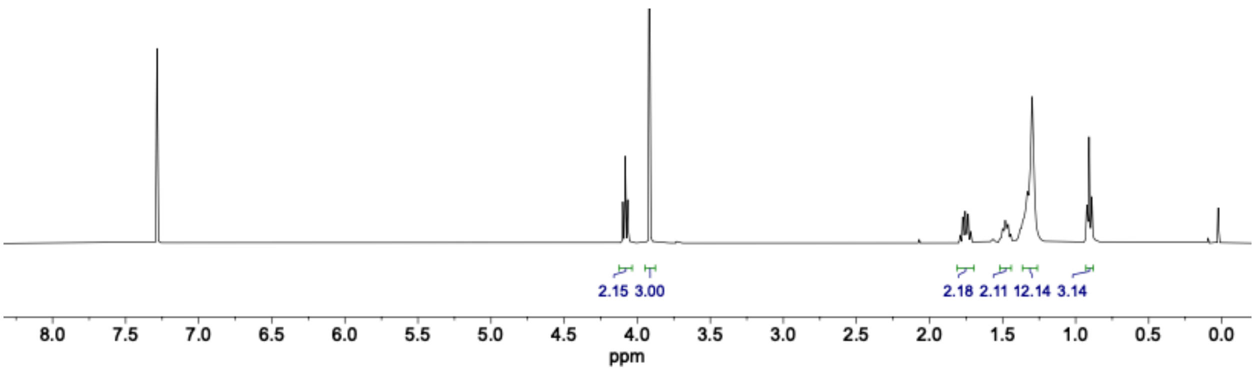

Figure S6. ^{1}H-NMR (400 MHz) spectrum of AcDOT-[CH_3-C_{10}]-Br_2 in $CDCl_3$.

Polymer Synthesis

PAcDOT[C_{10}]$_2$

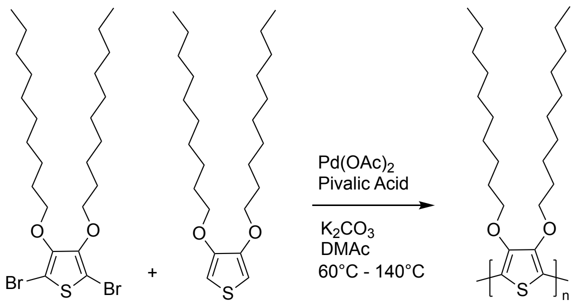

Palladium(II) acetate ($Pd(OAc)_2$, 5.7 mg, 25 μmol, 0.02 eq), pivalic acid (0.039 g, 0.38 mmol, 0.3 eq), anhydrous K_2CO_3 (0.435 g, 3.2 mmol, 2.5 eq) were added to a dry 50 mL round bottom flask with stir bar. AcDOT-[C_{10}]$_2$-Br_2 (0.699 g, 1.3 mmol, 1 eq) and AcDOT-[C_{10}]$_2$ (0.5 g, 1.3 mmol, 1 eq) were both added to the flask. Dimethylacetamide (DMAc) (0.2 M to total monomer, 12.6 mL) was added and the flask sealed with rubber septum. The contents were purged with argon. The flask was heated from 60 °C to 140 °C and reacted overnight. After cooling, the contents were diluted with a small amount of hot $CHCl_3$ and precipitated into methanol. The precipitate was loaded into a Soxhlet thimble and subsequently washed with methanol, acetone, hexane, toluene, and $CHCl_3$. All washings were done until the solvent ran clear or for two hours, whichever was

longest. Most of the polymer was collected in the $CHCl_3$ fraction, dried with rotary evaporation, reprecipitated into MeOH, then collected by filtration with a 45 μm pore size Nylon pad. The polymer was collected as dark maroon chunks ($CHCl_3$ fraction: 350 mg, 35 %). ^{1}H-NMR (400 MHz, $CDCl_3$, 25 °C) δ (ppm) 4.14 (4H, br), 1.93 (4H, br), 1.48 (4H, br), 1.29 (24H, br), 0.90 (6H, m). Anal. calcd. for $C_{24}H_{42}O_2S$ C 73.04, H 10.73, S 8.12, Found C 72.94, H 10.77, S 7.95. M_n = 14.6 kg/mol, Đ = 1.79, vs. PS in $CHCl_3$ at 40 °C vs. PS standards.

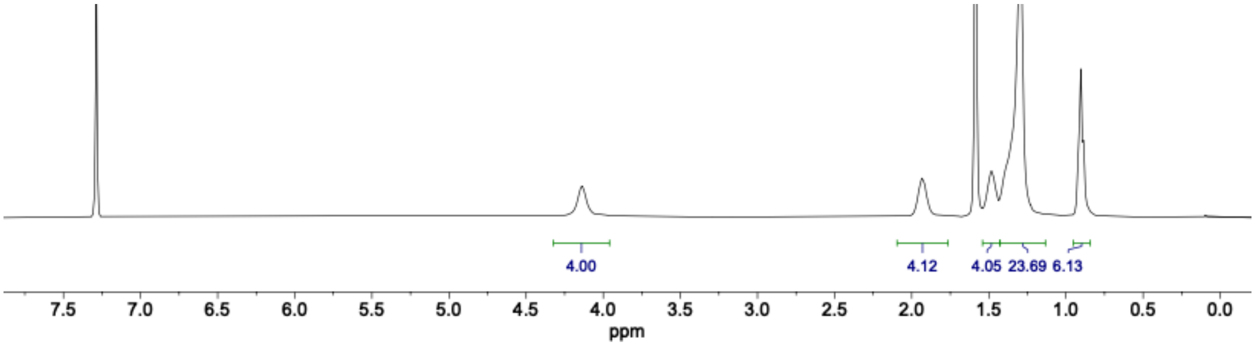

Figure S7. ^{1}H-NMR (400 MHz) spectrum of PAcDOT$[C_{10}]_2$ in $CDCl_3$.

<u>PAcDOT$[C_{10}]_2$-co-$[OE_3]_2$</u>

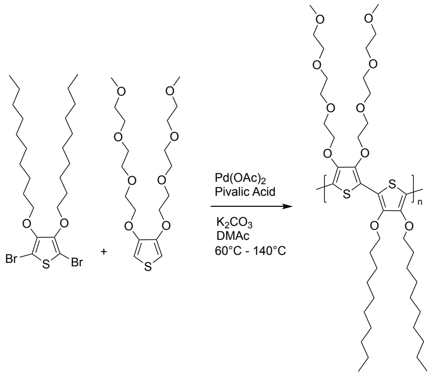

Palladium(II) acetate ($Pd(OAc)_2$, 5.5 mg, 24 μmol, 0.02 eq), pivalic acid (0.037 g, 0.37 mmol, 0.3 eq), anhydrous K_2CO_3 (0.422 g, 3.1 mmol, 2.5 eq) were added to a dry 50 mL round bottom flask with stir bar. AcDOT-$[C_{10}]_2$-Br_2 (0.679 g, 1.2 mmol, 1 eq) and AcDOT-$[OE_3]_2$ (0.5 g, 1.2 mmol, 1 eq) were both added to the flask. DMAc (0.2 M to total monomer, 12.2 mL) was added and the

flask sealed with rubber septum. The contents were purged with argon. The flask was heated from 60 °C to 140 °C and reacted overnight. After cooling, the contents were diluted with a small amount of $CHCl_3$ and precipitated into methanol. The precipitate was loaded into a Soxhlet thimble and subsequently washed with methanol, acetone, ethyl acetate, and $CHCl_3$. All washings were done until the solvent ran clear or for two hours, whichever was longest. Polymer was collected in the ethyl acetate (300 mg) and $CHCl_3$ washes (420 mg). Only the $CHCl_3$ fraction was used in this study, although the ethyl acetate fraction was pure by NMR and elemental analysis with a molecular mass roughly 20 % lower than the $CHCl_3$ fraction. The $CHCl_3$ fraction was dried with rotary evaporation, reprecipitated into MeOH, then collected by filtration with a 45 µm pore size Nylon pad. The polymer was collected as dark purple chunks ($CHCl_3$ fraction: 420 mg, 42 %). ^{1}H-NMR (400 MHz, $CDCl_3$, 25 °C) δ (ppm) 4.35 (4H, br), 4.13 (4H, br), 3.92 (4H, br), 3.72 (4H, br), 3.65 (8H, br), 3.53 (4H, br), 3.37 (6H, s), 1.92 (4H, br), 1.48 (4H, br), 1.29 (24H, br), 0.90 (6H, t, J = 6.6 Hz). Anal. calcd. for $C_{42}H_{72}O_{10}S_2$ C 62.97, H 9.06, S 8.00, Found C 62.96, H 9.12, S 7.90. M_n = 30.0 kg/mol, Đ = 1.75, vs. PS in $CHCl_3$ at 40 °C vs. PS standards.

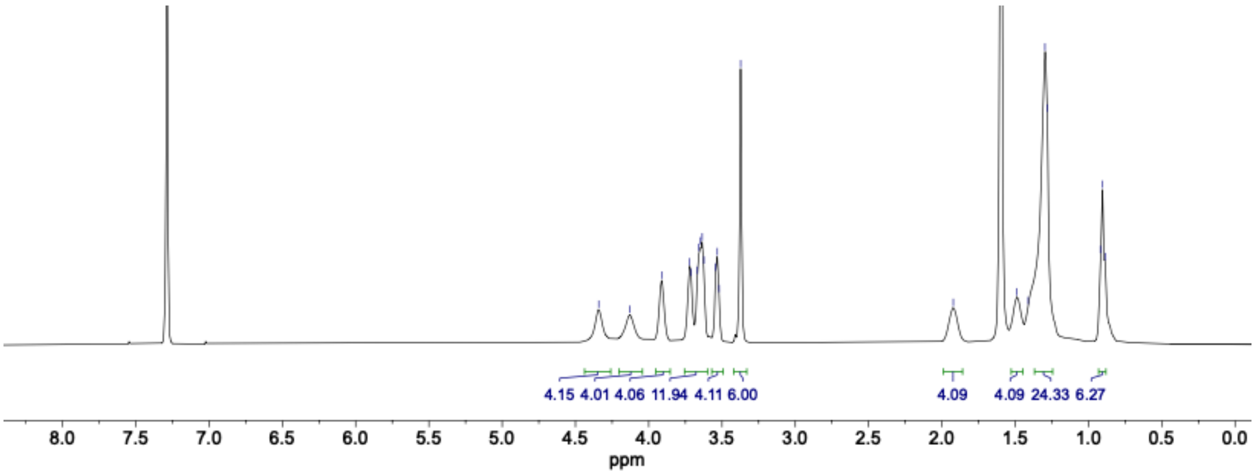

Figure S8. ^{1}H-NMR (400 MHz) spectrum of PAcDOT[C_{10}]$_2$-co-[OE_3]$_2$ in $CDCl_3$.

<u>PAcDOT[CH_3-C_{10}]</u>

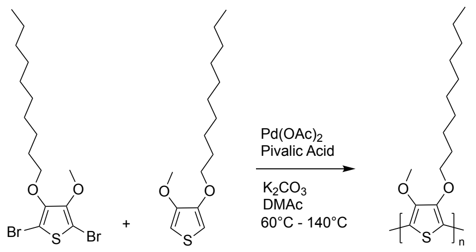

Palladium(II) acetate ($Pd(OAc)_2$, 8.3 mg, 37 µmol, 0.02 eq), pivalic acid (0.057 g, 0.55 mmol, 0.3 eq), anhydrous K_2CO_3 (0.64 g, 4.6 mmol, 2.5 eq) were added to a dry 50 mL round bottom flask with stir bar. AcDOT-[CH_3-C_{10}]-Br_2 (0.792 g, 1.85 mmol, 1 eq) and AcDOT-[CH_3-C_{10}] (0.5 g, 1.85 mmol, 1 eq) were both added to the flask. DMAc (0.2 M to total monomer, 18.5 mL) was added and the flask sealed with rubber septum. The contents were purged with argon. The flask was heated from 60 °C to 140 °C and reacted overnight. After cooling, the contents were diluted with a small amount of $CHCl_3$ and precipitated into methanol. The precipitate was loaded into a Soxhlet thimble and subsequently washed with methanol, acetone, ethyl acetate, hexanes, and $CHCl_3$. The $CHCl_3$ fraction was dried with rotary evaporation, reprecipitated into MeOH, then collected by filtration with a 45 µm pore size Nylon pad. The polymer was collected as dark maroon chunks ($CHCl_3$ fraction: 350 mg, 35 %). ^{1}H-NMR (400 MHz, $CDCl_3$, 25 °C) δ (ppm) 4.18 (2H, br), 4.00 (3H, s), 1.96 (2H, br), 1.51 (2H, br), 1.30 (12H, br), 0.91 (3H, br). Anal. calcd. for $C_{15}H_{24}O_2S$ C 67.12, H 9.01, S 11.94, Found C 66.89, H 9.07, S 11.76. M_n = 10.6 kg/mol, Đ = 1.56, vs. PS in $CHCl_3$ at 40 °C vs. PS standards.

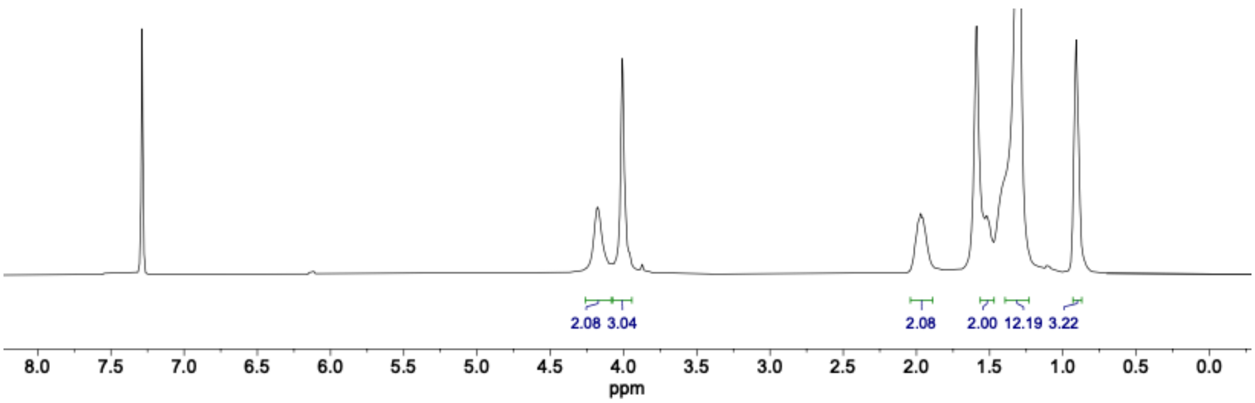

Figure S9. ^{1}H-NMR (400 MHz) spectrum of PAcDOT[CH_3-C_{10}] in $CDCl_3$.

<u>PAcDOT[CH_3-C_{10}]-co-[CH_3-OE_3]</u>

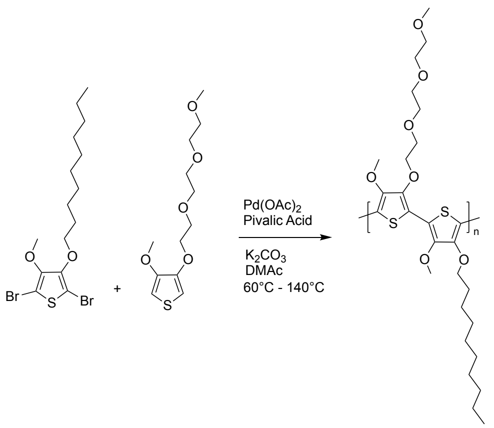

Palladium(II) acetate ($Pd(OAc)_2$, 16 mg, 72 μmol, 0.02 eq), pivalic acid (0.11 g, 1.1 mmol, 0.3 eq), anhydrous K_2CO_3 (1.25 g, 9.0 mmol, 2.5 eq) were added to a dry 100 mL round bottom flask with stir bar. AcDOT-[CH_3-C_{10}]-Br_2 (1.55 g, 3.6 mmol, 1 eq) and AcDOT-[CH_3-OE_3] (1.0 g, 3.6 mmol, 1 eq) were both added to the flask. DMAc (0.2 M to total monomer, 36 mL) was added and the flask sealed with rubber septum. The contents were purged with argon. The flask was heated from 60 °C to 140 °C and reacted overnight. After cooling, the contents were diluted with a small amount of $CHCl_3$ and precipitated into methanol. The precipitate was loaded into a Soxhlet thimble and subsequently washed with methanol, acetone, hexanes, and $CHCl_3$. The $CHCl_3$ fraction was

dried with rotary evaporation, reprecipitated into MeOH, then collected by filtration with a 45 µm pore size Nylon pad. The polymer was collected as dark maroon chunks ($CHCl_3$ fraction: 1.39 g, 69 %). ^{1}H-NMR (400 MHz, $CDCl_3$, 25 °C) δ (ppm) 4.35 (2H, br), 4.17 (2H, br), 4.1-3.9 (8H, m), 3.85-3.63 (6H, m), 3.56 (2H, br), 3.39 (3H, s), 1.96 (2H, br), 1.51 (2H, br), 1.31 (12H, br), 0.90 (3H, br). Anal. calcd. for $C_{27}H_{42}O_7S_2$ C 59.75, H 7.80, S 11.81, Found C 60.89, H 8.12, S 11.02. M_n = 14.0 kg/mol, Đ = 2.15, vs. PS in $CHCl_3$ at 40 °C vs. PS standards.

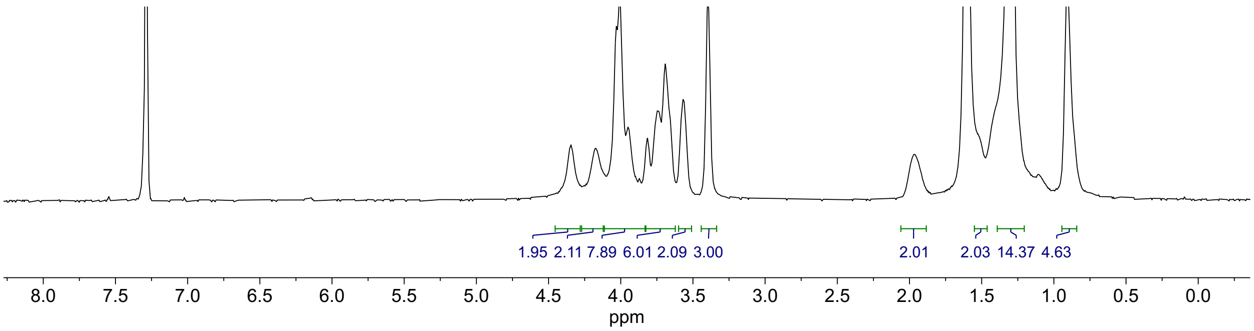

Figure S10. ^{1}H-NMR (400 MHz) spectrum of PAcDOT[CH_3-C_{10}]-co-[CH_3-OE_3] in $CDCl_3$. We note the slight increase of alkyl intensity near 1.4 to 0.8 ppm may be attributed to 1-decanol impurity.

Supplemental Figures

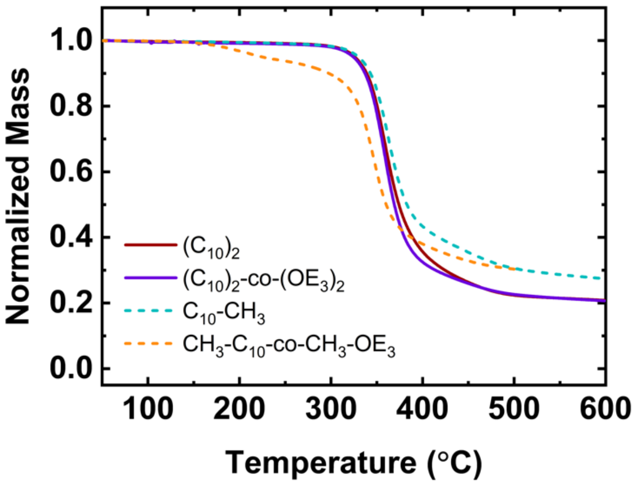

Figure S11. Thermogravimetric analysis performed from 50 to 600 °C at a ramp rate of 20 °C/min under nitrogen atmosphere in alumina crucibles. Samples were dried at 80 °C at 10 torr overnight. There is an initial loss of mass below 200 °C for [CH_3-C_{10}-co-CH_3-OE_3], which leads to 5 % mass loss near 230 °C and 15 % near 300 °C. This is followed by a similar mass loss observed for other PAcDOTs. Despite the slight mass loss, no observable change in the π-π* and diffraction intensity leads us to hypothesize that the backbone is unaffected.

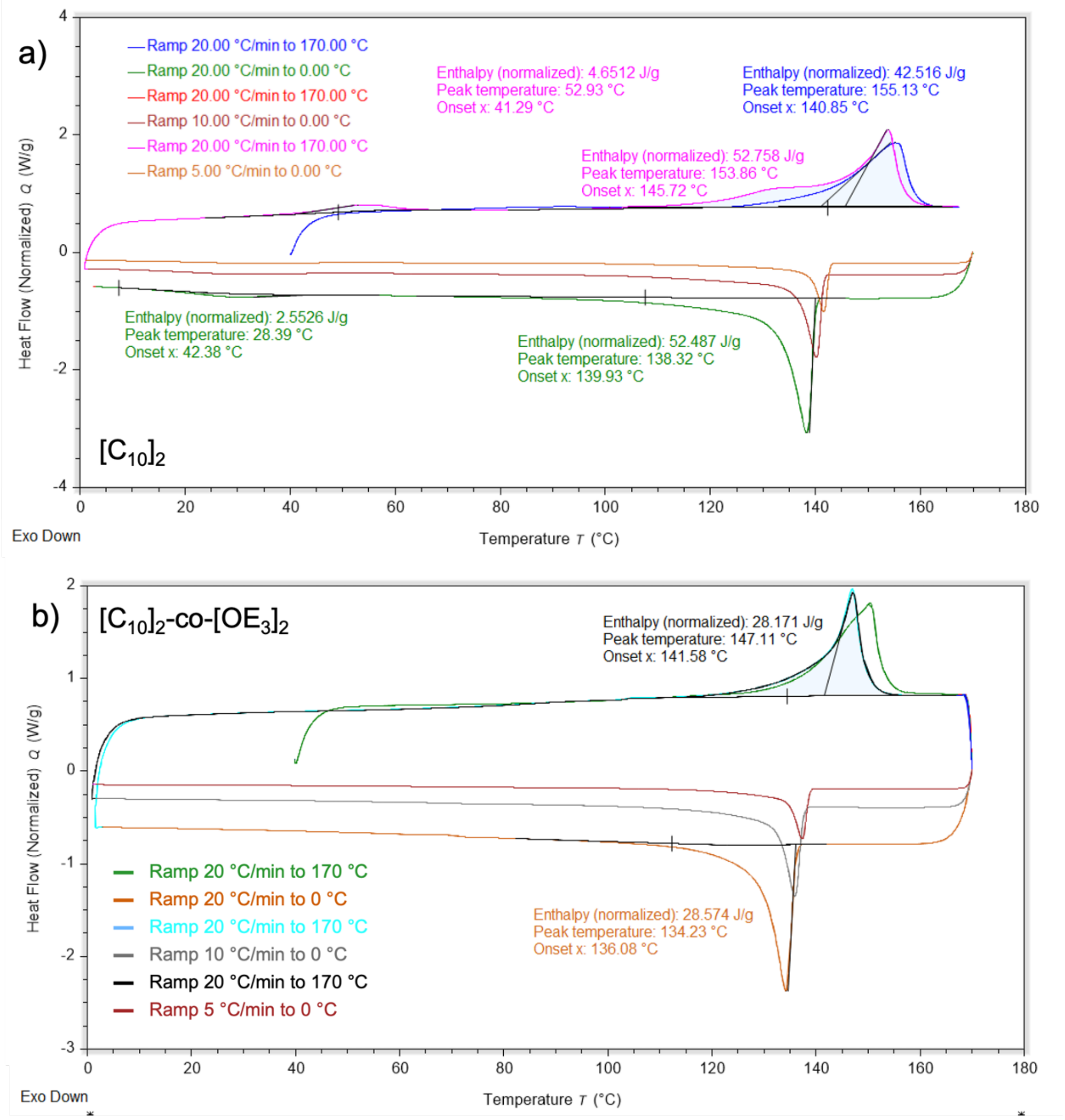

Figure S12. Differential scanning calorimetry (DSC) of a) $[C_{10}]_2$ and b) $[C_{10}]_2$-co-$[OE_3]_2$ regio-symmetric PAcDOTs done in T-Zero aluminum pans. The heating and cooling rates are indicated in each plot. The exothermic direction is down.

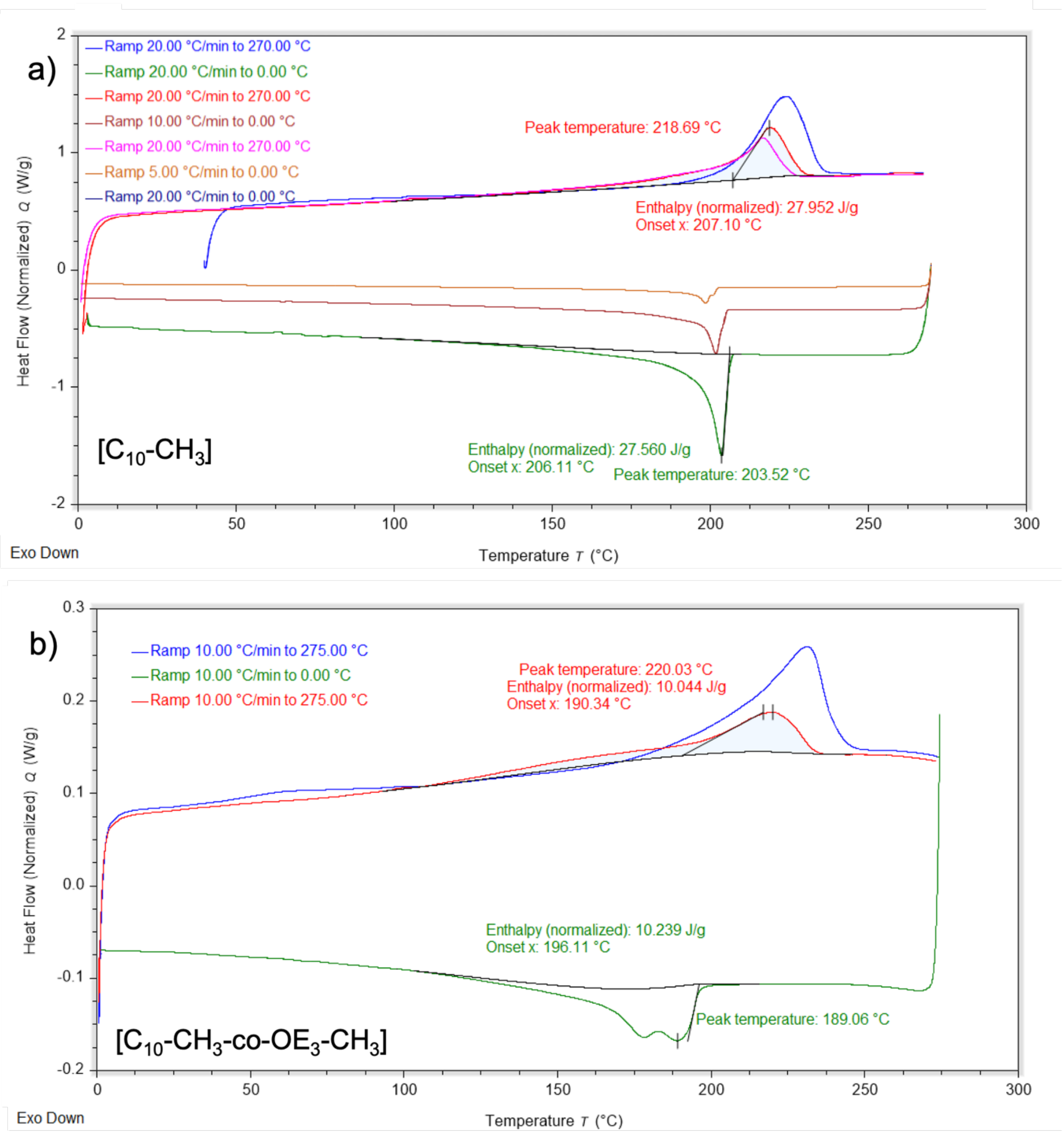

Figure S13. DSC of a) [CH_3-C_{10}] and b) [CH_3-C_{10}-co-CH_3-OE_3] regio-asymmetric PAcDOTs done in T-Zero aluminum pans. The heating and cooling rates are indicated in each plot. The exothermic direction is down.

Table S1. Peak Melting and Crystallization Temperatures of PAcDOT Powders

Polymer	$T_{1, heat}$ (°C)	$T_{1, cool}$ (°C)	$T_{2, heat}$ (°C)	$T_{2, cool}$ (°C)	$T_{3, heat}$ (°C)	$T_{3, cool}$ (°C)
$[C_{10}]_2$	53	28	133	Convoluted with T_3	154	138
$[C_{10}]_2$-co-$[OE_3]_2$			Convoluted with T_3	Convoluted with T_3	147	134
$[CH_3$-$C_{10}]$					219	204
$[CH_3$-$C_{10}]$-co-CH_3-OE_3					220	189

Table S2. Summary Thermodynamic Properties of PAcDOTs[1]

	$[C_{10}]_2$	$[C_{10}]_2$-co-$[OE_3]_2$	$[CH_3$-$C_{10}]$	$[CH_3$-$C_{10}]$-co-CH_3-OE_3	P3HT[2]
$T_{m, peak}$ (K)	427	420	492	493	545
ΔH_f (J/g)	52	28	28	10	49
ΔH_f (kJ/mol)	20	12	7.6	2.7	8.1
ΔS_f (J/K mol)	47	28	15	5.5	15

1. All quantities per mol thiophene (backbone mobile unit)
2. All values for P3HT are extrapolated to the 100 % crystalline polymer in the infinite chain limit as described in Snyder et al.[1]

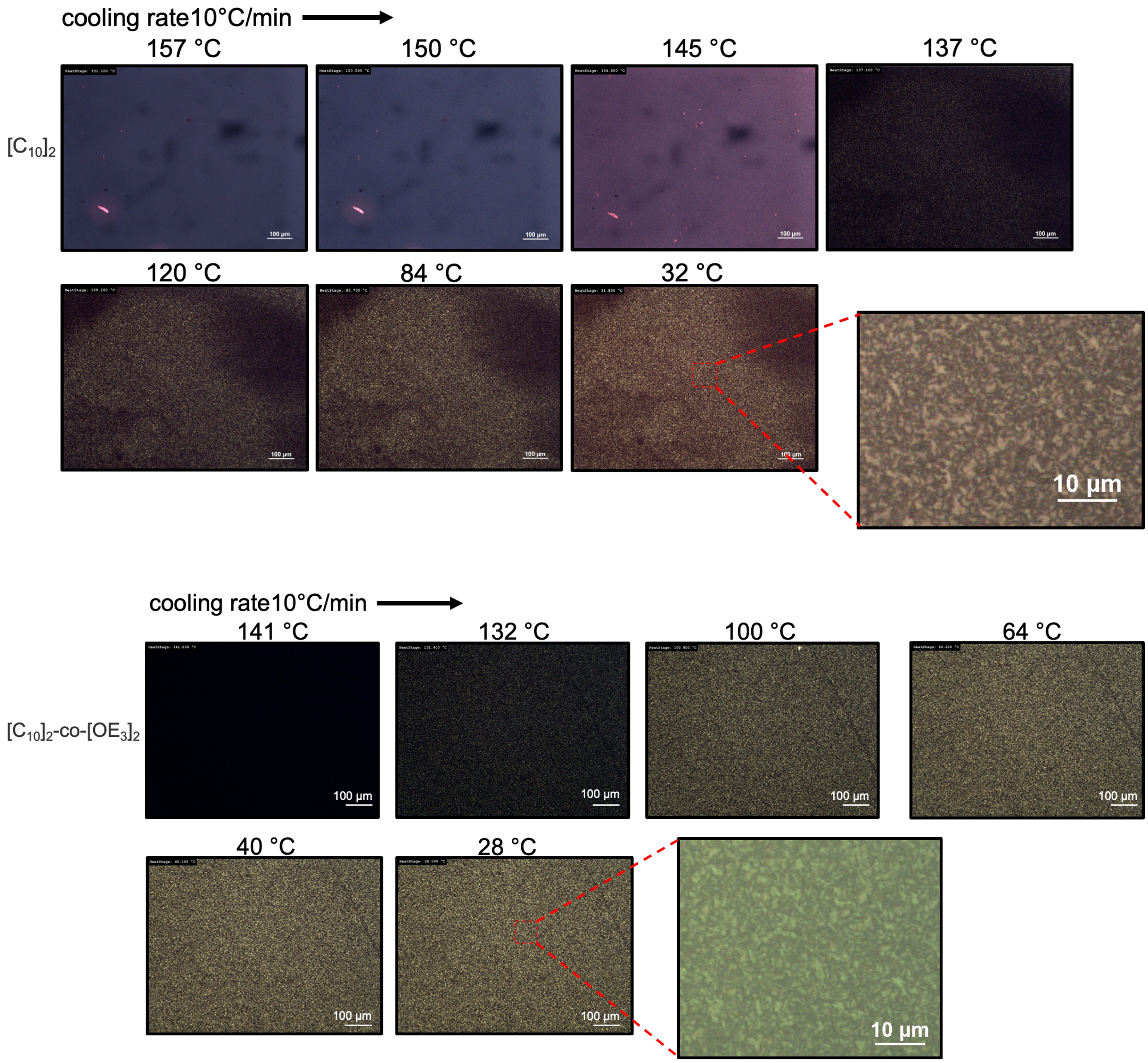

Figure S14. Crossed polarization optical microscopy (CPOM) images showing the nucleation and growth of birefringent crystals of melt-pressed regio-symmetric PAcDOTs. Powders are pressed between microscope slides, fully melted, then imaged during cooling from the melt. Many nucleation points seem to exist, which grow into impinging birefringent crystals. The exact growth mechanism or crystal morphology is uncertain.

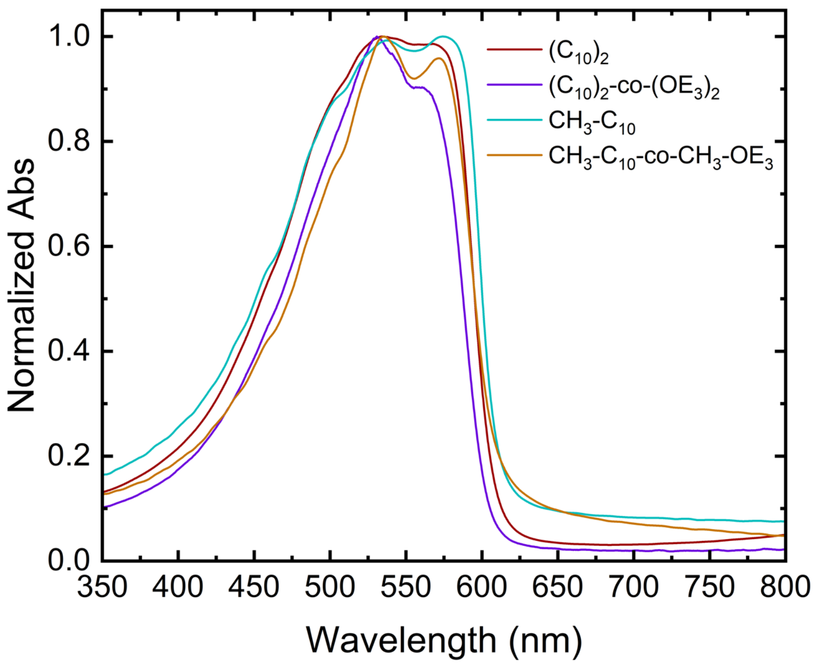

Figure S15. Solution UV-vis spectra measured with 0.1 mm path length cuvettes at a polymer concentration of 5 mg/mL in $CHCl_3$.

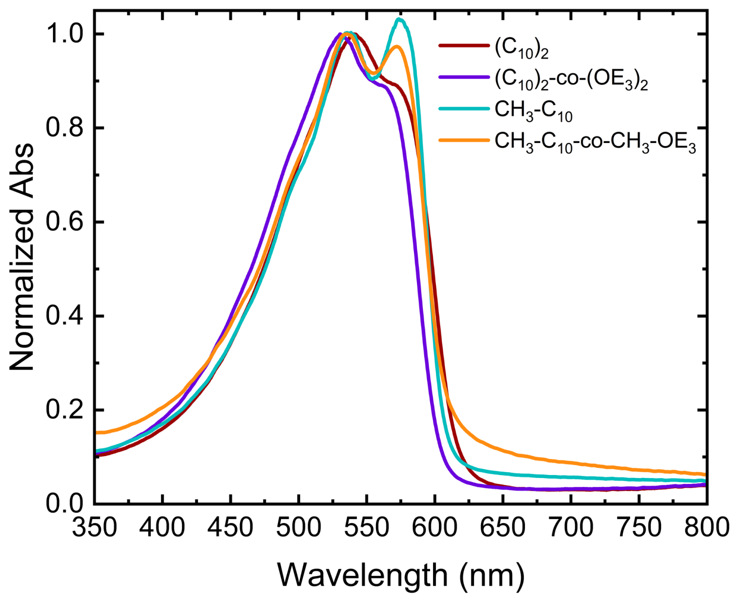

Figure S16. UV-Vis absorption spectra of 0.025 mg/mL polymer solutions in $CHCl_3$ using a standard 1 cm path length cuvette.

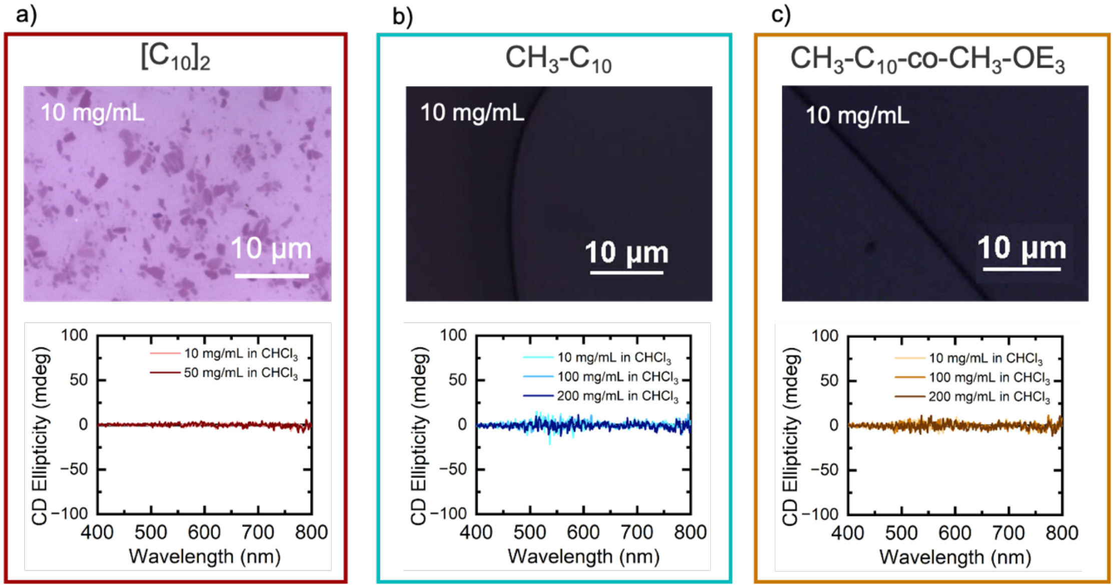

Figure S17. a) $CHCl_3$ solution CPOM of $[C_{10}]_2$ and the corresponding circular dichroism spectrum. The non-polar, ordered side-chains result in micron-scale aggregates. b and c) Drying meniscus of a 10 mg/mL $CHCl_3$ solution of regio-asymmetric polymers. CPOM and the corresponding circular dichroism plots as a function of wavelength for b) $[CH_3\text{-}C_{10}]$ and c) $[CH_3\text{-}C_{10}]\text{-co-}CH_3\text{-}OE_3]$. CPOM images show the drying meniscus, allowing for the potential identification of mesophases due to the drying meniscus exhibited concentrations from 10 mg/mL to the bulk solid. Both drying meniscuses are isotropic under crossed polarizers, indicating the absence of any liquid crystal mesophases. Circular dichroism indicates solutions have no measurable ellipticity, and thus no chiral emergence.

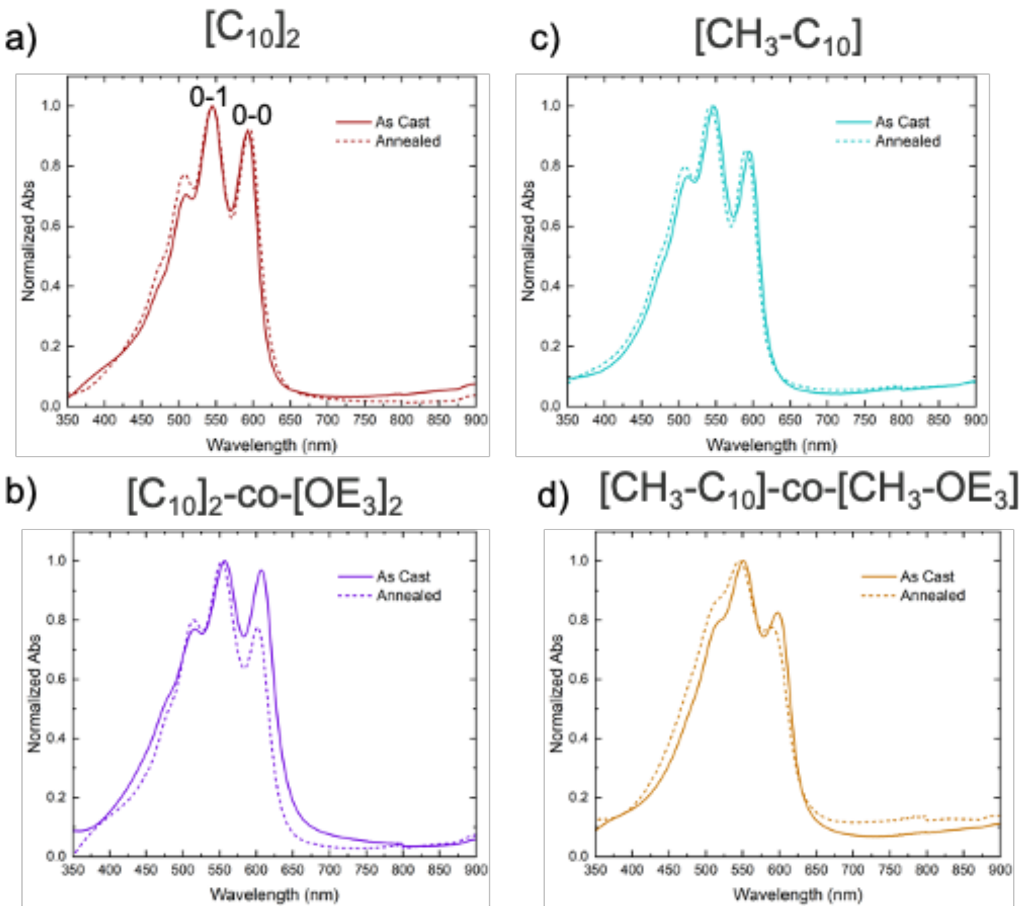

Figure S18. Normalized thin film UV-Vis of as-cast and annealed a) $[C_{10}]_2$, b) $[C_{10}]_2$-co-$[OE_3]_2$, c) $[CH_3$-$C_{10}]$, and d) $[CH_3$-C_{10}-co-CH_3-$OE_3]$ PAcDOTs. The most significant difference occurs for $[C_{10}]_2$-co-$[OE_3]_2$, where as-cast films have a stronger absorption of the vibronic shoulder, suggesting weaker interacting chains (higher 0-0 to 0-1 ratio) and possibly stronger intra-chain coherence.

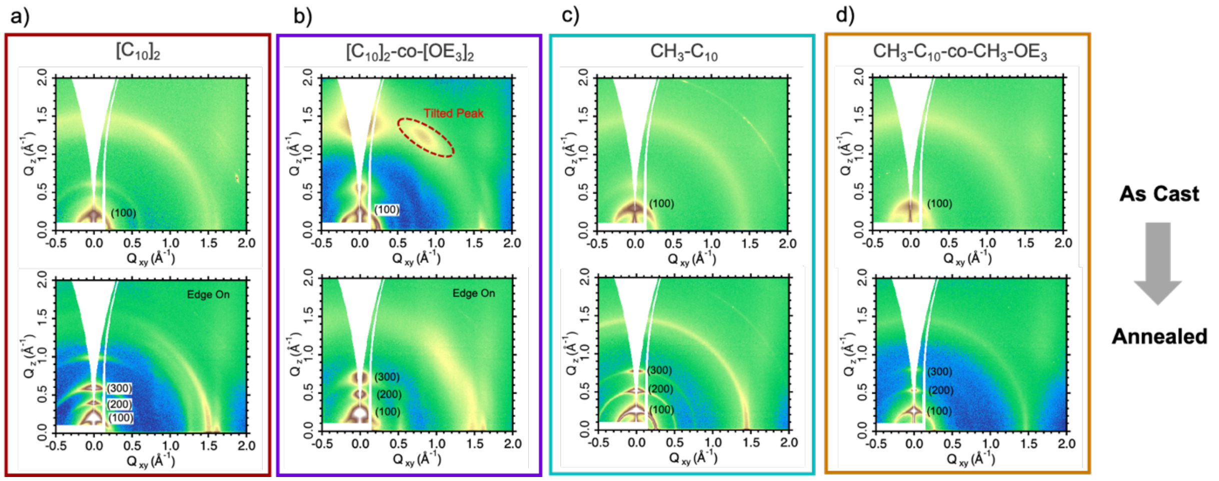

Figure S19. Ex situ 2D grazing-incidence wide-angle X-ray scattering (GIWAXS) patterns of as-cast and annealed films of a) $[C_{10}]_2$, b) $[C_{10}]_2$-co-$[OE_3]_2$, c) $[CH_3$-$C_{10}]$, and d) $[CH_3$-C_{10}-co-CH_3-$OE_3]$ PAcDOTs. As cast films show non-equilibrium morphologies, with the most unique being a chiral phase in as-cast $[C_{10}]_2$-co-$[OE_3]_2$. Regio-symmetric polymers become more edge on with annealing, while all polymers show enhanced lamellar ordering.

Table S3. Ex Situ Out-of-Plane (100) d-spacings for As-Cast and Annealed PAcDOT Films

PAcDOT	Film State	(100) d-spacing (Å)
$[C_{10}]_2$	As Cast	32.0
	Annealed	31.6
$[C_{10}]_2$-co-$[OE_3]_2$	As Cast	27.4
	Annealed	25.4
$[CH_3$-$C_{10}]$	As Cast	22.7
	Annealed	24.3
$[CH_3$-$C_{10}]$-co-CH_3-OE_3	As Cast	20.5
	Annealed	23.2

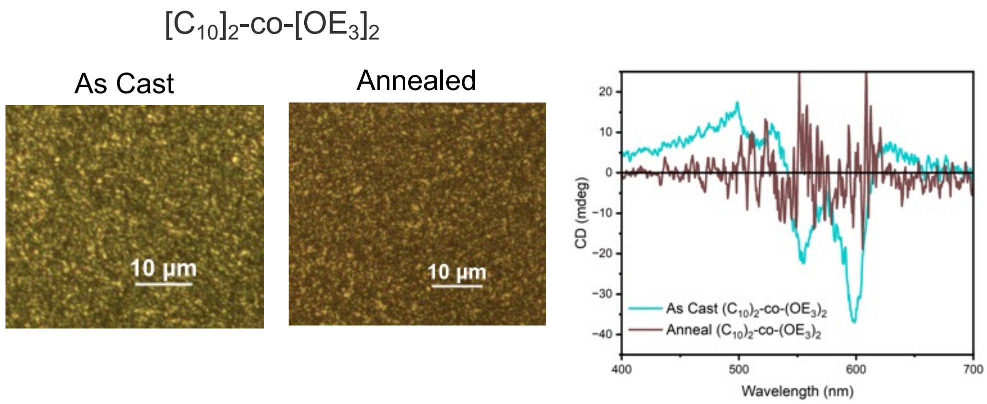

Figure S20. CPOM images and CD signal of as-cast and annealed $[C_{10}]_2$-co-$[OE_3]_2$ blade coated films. Birefringence is still visible after annealing, although the clear absence of CD signal indicates the loss of chirality.

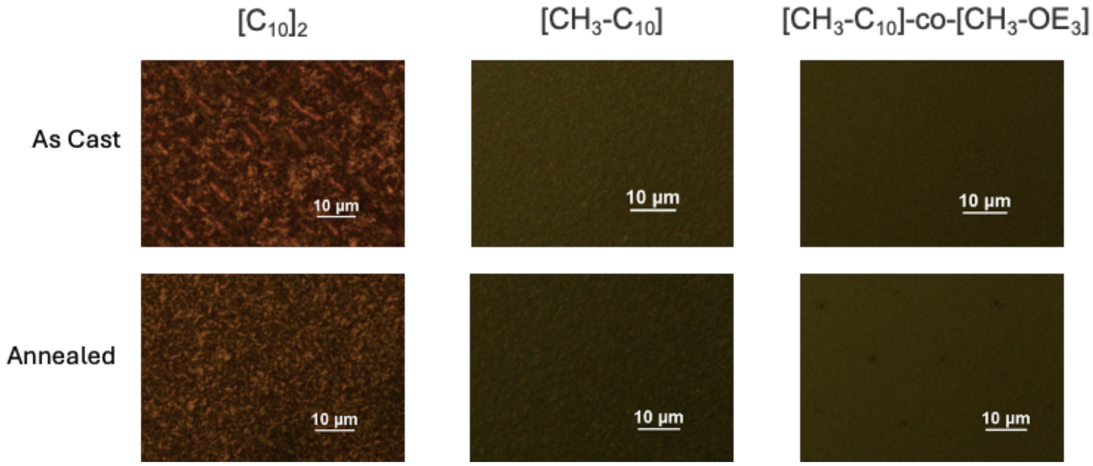

Figure S21. CPOM images of as-cast and annealed blade coated films. Regio-symmetric $[C_{10}]_2$ is birefringent with clear texture, although it is not chiral in either state. Regio-asymmetric side-chain substitution results in no birefringence or observed texture.

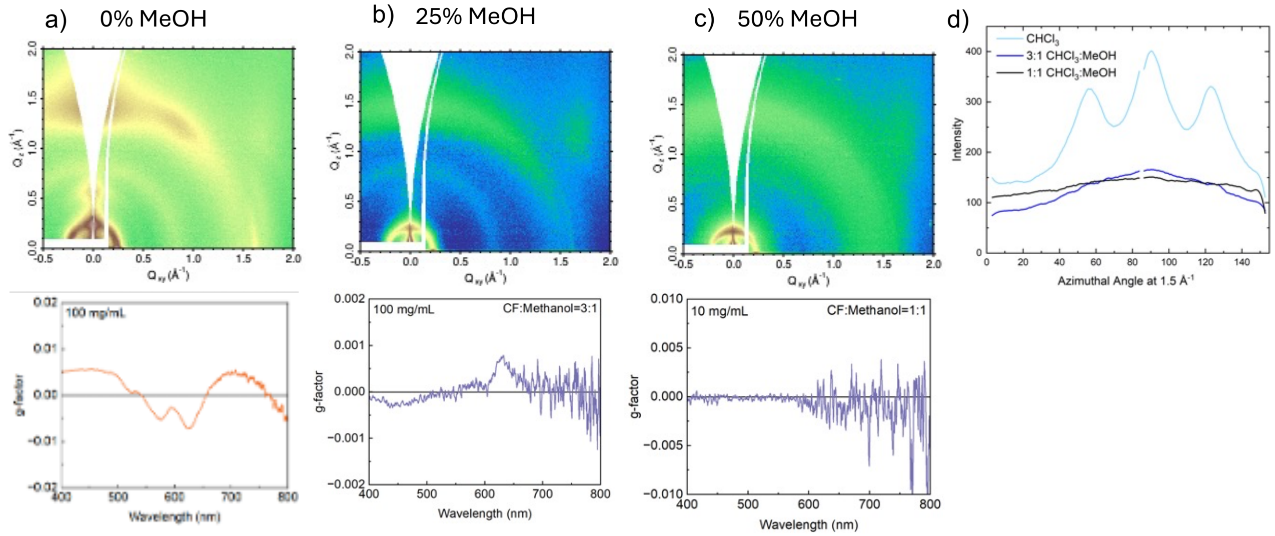

Figure S22. 2D GIWAXS images and measured g-factor of $[C_{10}]_2$-co-$[OE_3]_2$ blade coated from a) 100 % $CHCl_3$, b) 25 % by volume methanol (MeOH) in $CHCl_3$, and c) 50 % by volume MeOH in $CHCl_3$. d) The corresponding line cuts as function of azimuthal angle at 1.5 $Å^{-1}$ and measured g-factor show how the addition of MeOH as a poor solvent to the polymer solution leads to a loss of tilted peaks and chirality after blade coating.

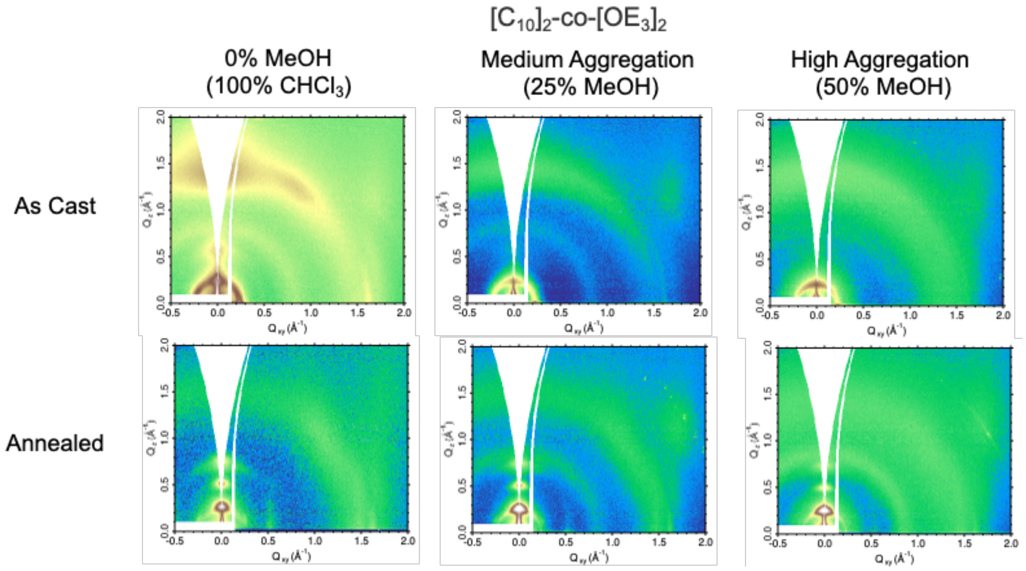

Figure S23. 2D GIWAXS images of $[C_{10}]_2$-co-$[OE_3]_2$ blade coated from 100 % $CHCl_3$, 3:1 $CHCl_3$:MeOH, and 1:1 $CHCl_3$:MeOH. Although the as-cast morphologies vary depending on solvent composition, the annealed structures are extremely similar in morphology and peak positions. This indicates that melting the polymer and slowly cooling allows the film to reach an equilibrium morphology that is independent of processing solvent.

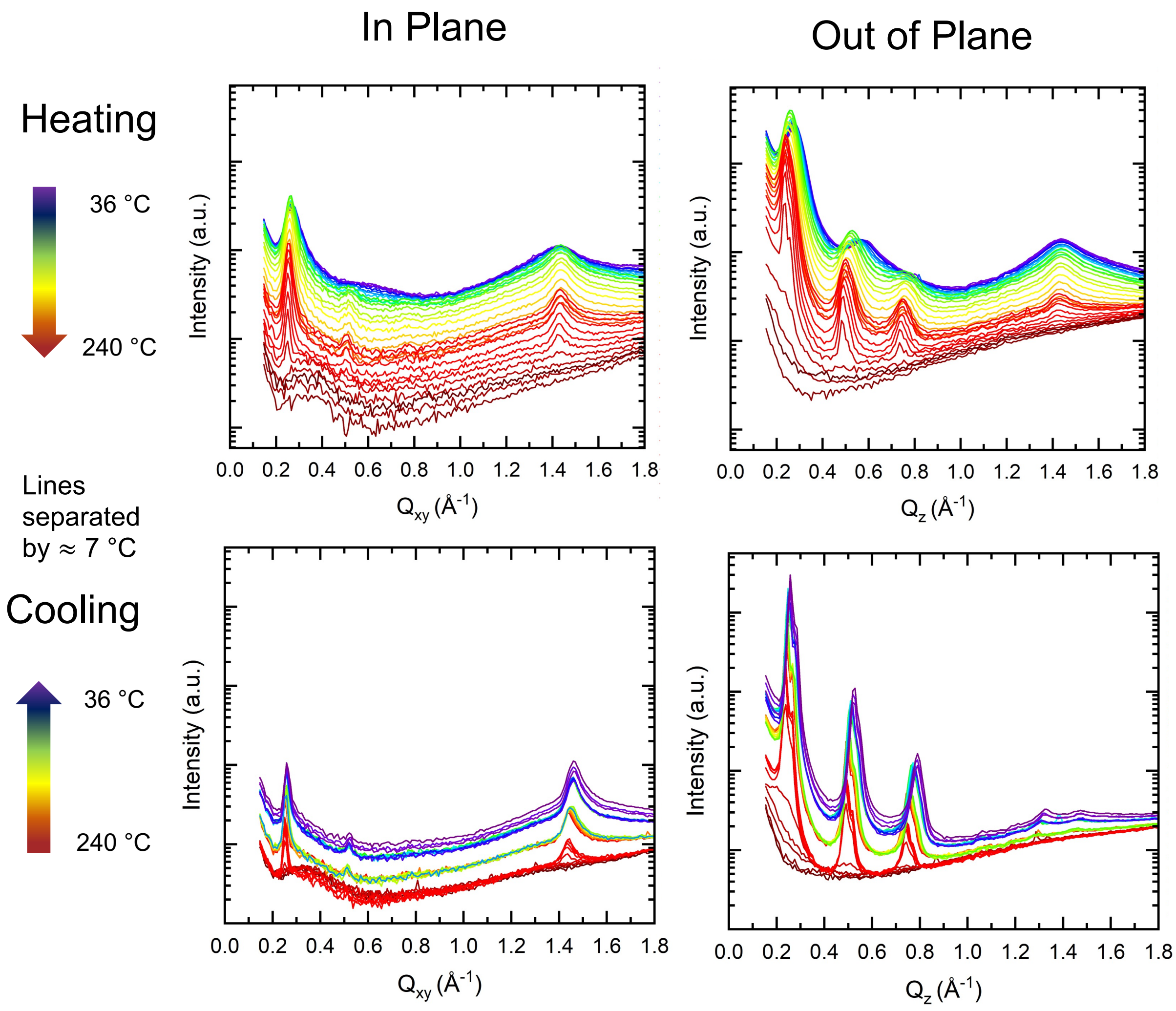

Figure S24. In situ thermal GIWAXS linecuts showing the evolution of ordered features as a function of temperature for regio-asymmetric [CH_3-C_{10}]. Every line is separated by approximately 7 °C. As the film is heated, the FWHM of the peaks narrow and lamellae expand (shift to lower q). At temperatures near T_m and above, out of plane lamellae reflections up to (300) becomes clearly visible and the relative order increases. Once cooled near room temperature, the lamellae are still expanded compared to initial as-cast film. Splitting of the (100) apparent in the cooling is due to the second channel (reflection + diffraction) of the distorted wave Born approximation.

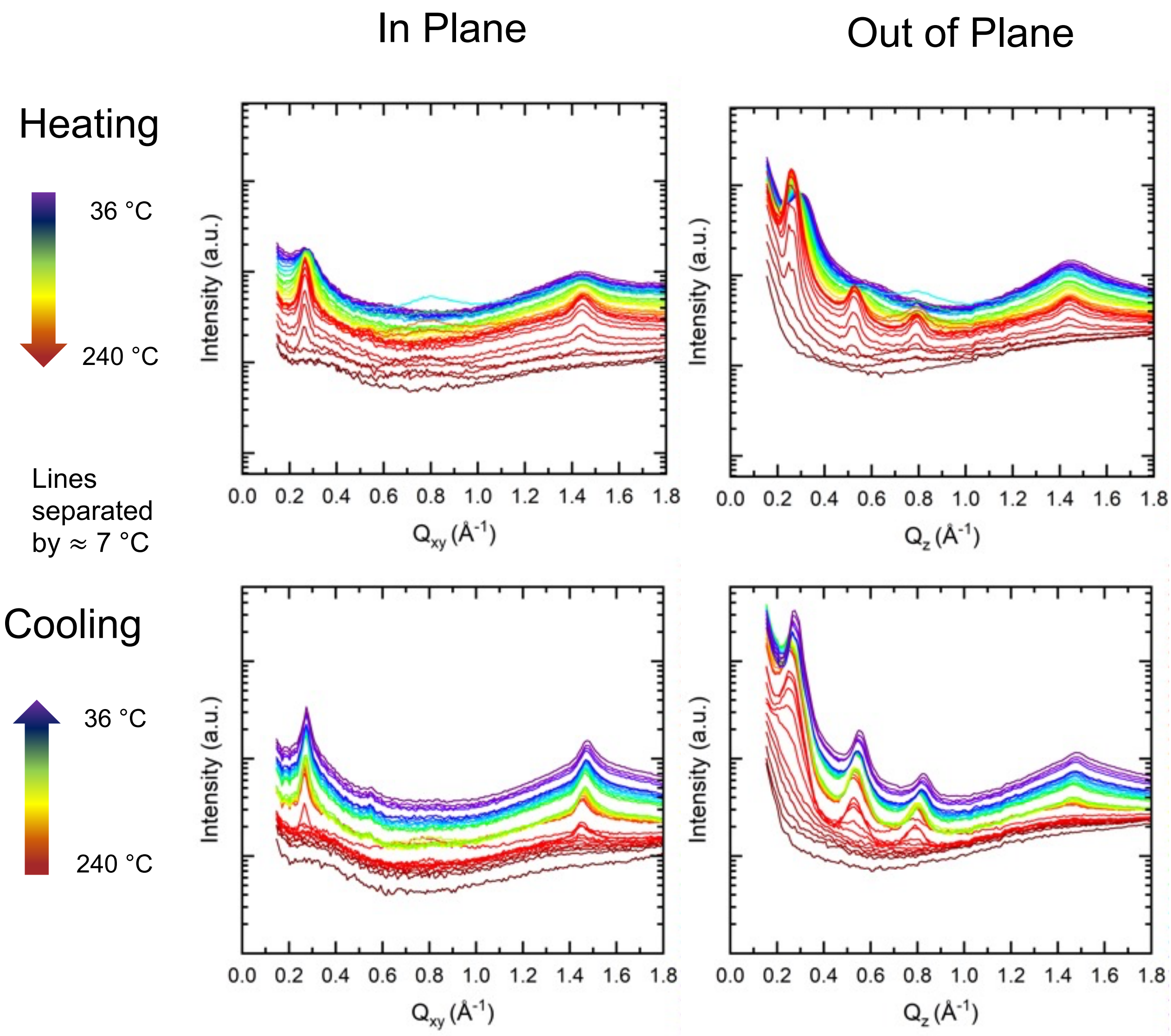

Figure S25. In situ thermal GIWAXS linecuts showing the evolution of ordered features as a function of temperature for regio-asymmetric [CH_3-C_{10}]-co-[CH_3-OE_3] film. As the film is heated, the FWHM of the peaks narrow and lamellae expand (shift to lower q). At temperatures near and above T_m , the (300) becomes clearly visible and the relative order increases. Once cooled near room temperature, the lamellae are still expanded compared to initial as-cast film.

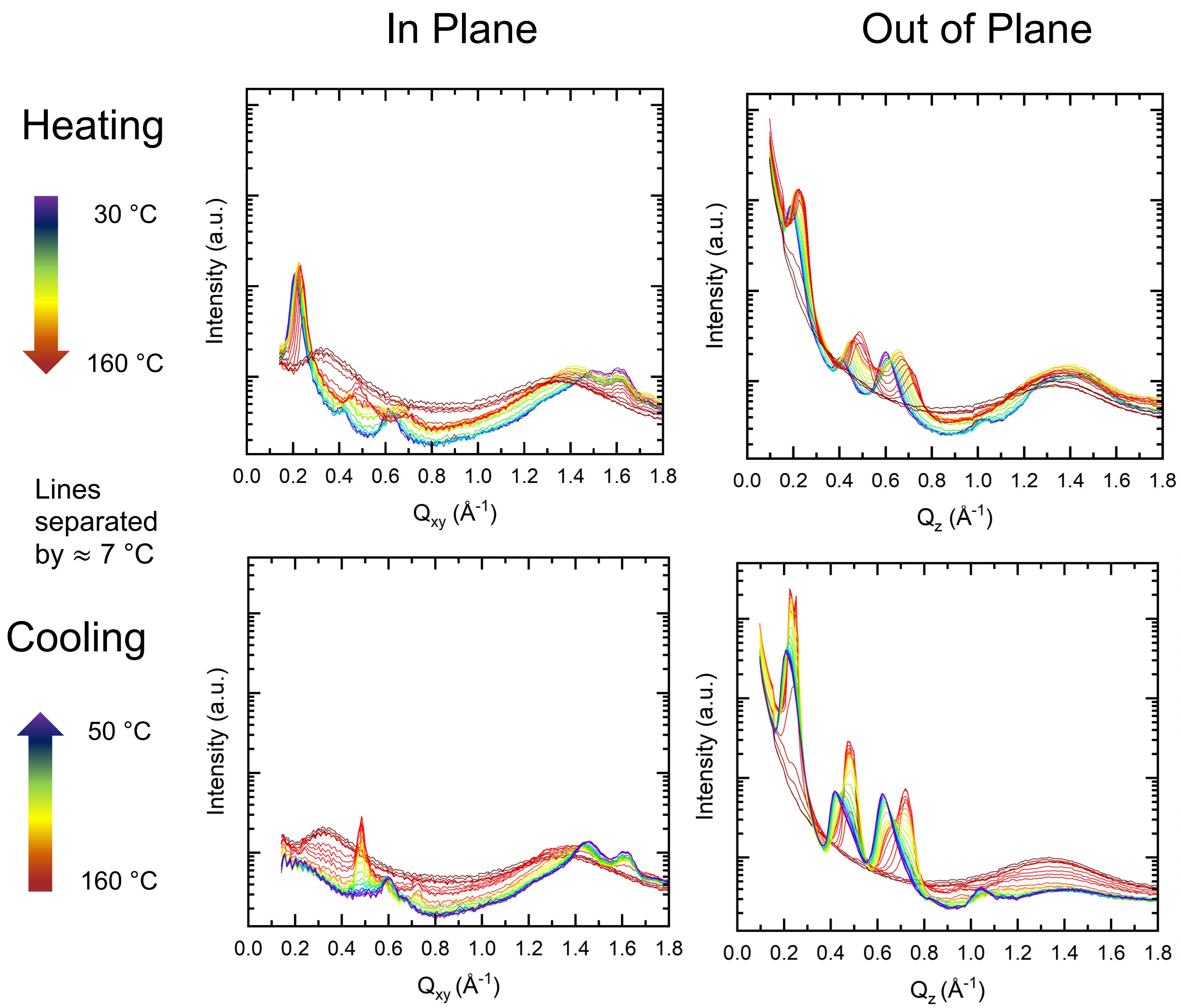

Figure S26. In situ thermal GIWAXS linecuts showing the evolution of ordered features as a function of temperature for regio-symmetric $[C_{10}]_2$ film. Understanding the structural evolution is complicated by the presence of multiple distinct phases. Upon heating from the as-cast state, the lamellae very slightly expand, although the overall morphology remains largely unchanged. Near 80 °C a phase change occurs as evidenced by an isosbestic point, clearly seen in the out-of-plane (300) diffraction. After melting and beginning to cool, a 3D ordered crystal, evidenced by mixed index diffraction peaks appears. Again, an isosbestic point appears near 81 °C, as the film transitions to a second phase with absence of 3D order (loss of mixed index peaks) and a larger d_{100}. This suggests that ordering of the side chains at lower temperature disrupts the 3D order of the high temperature, (80 to 140) °C phase.

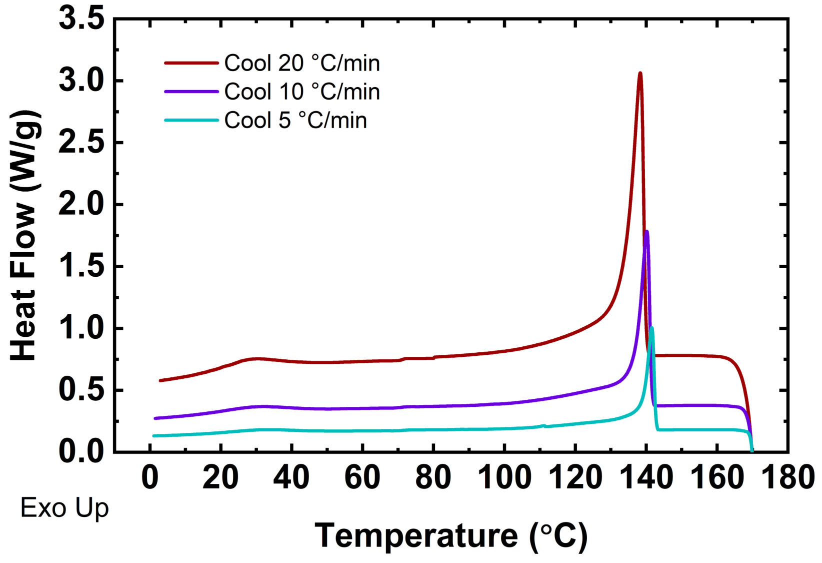

Figure S27. Cooling $[C_{10}]_2$ from the melt at different rates results in varying levels of undercooling, indicative of a nucleation and growth crystallization process, consistent with the 3D crystal phase observed in GIWAXS.

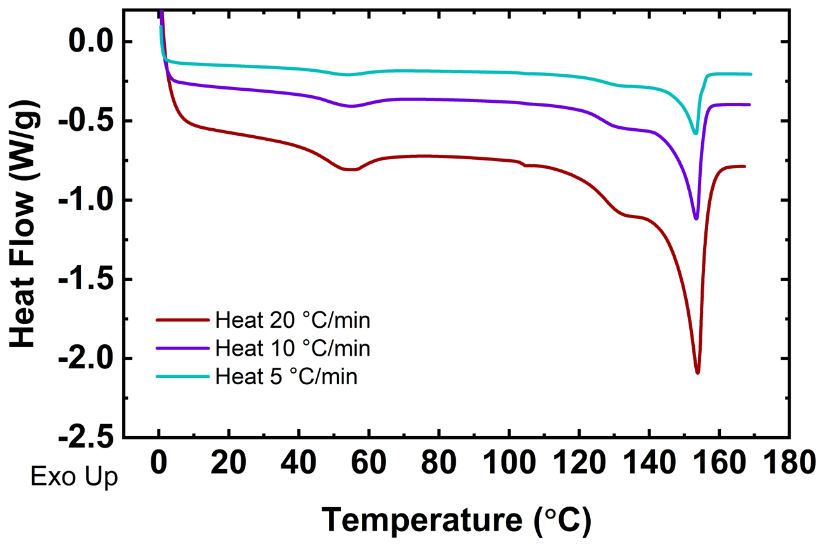

Figure S28. Heating $[C_{10}]_2$ from the low temperature crystalline phase at different rates results in little superheating difference. This is indicative of a flexible backbone and points to the phase transitions not being thermotropic liquid crystalline in nature.

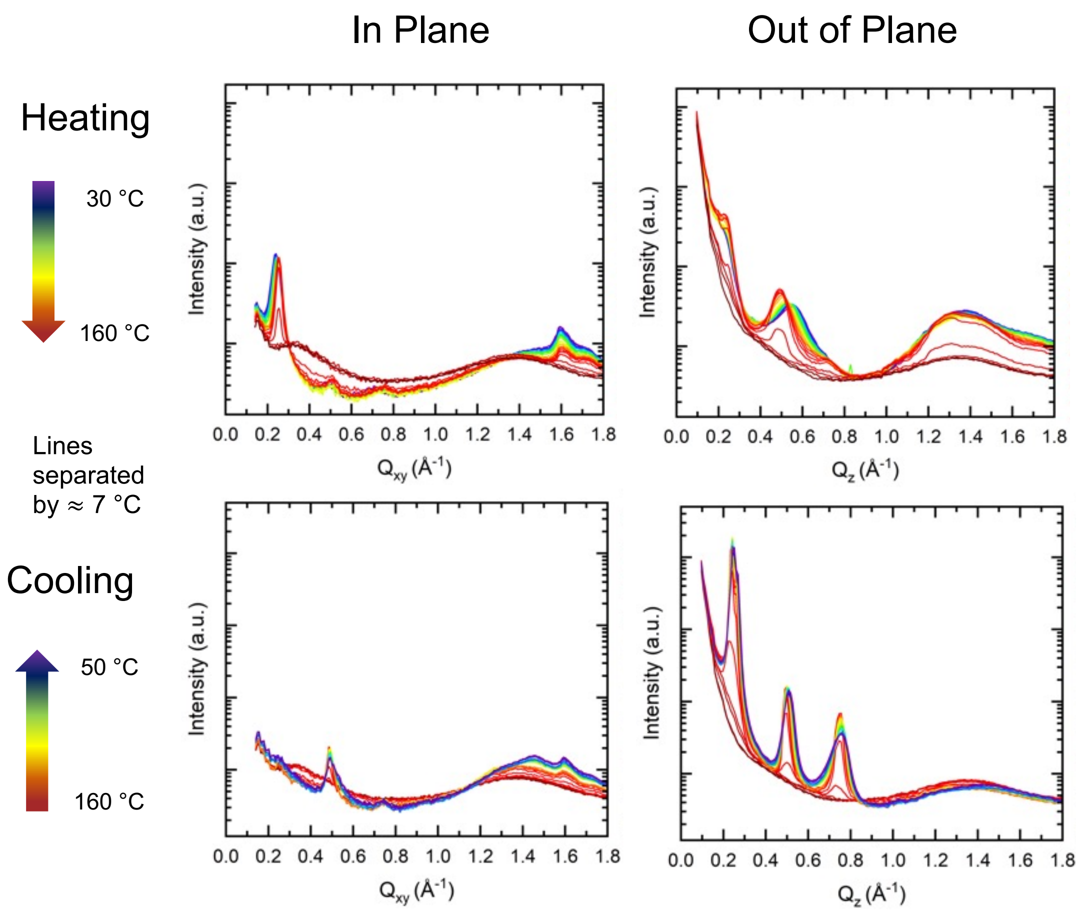

Figure S29. In situ thermal GIWAXS linecuts showing the evolution of ordered features as a function of temperature for regio-symmetric $[C_{10}]_2$-co-$[OE_3]_2$ film. After melting and beginning to cool, a similar pattern with mixed diffraction peaks forms near 117 °C. Unlike $[C_{10}]_2$, there is no second phase below the 3D crystal. The persistence of the 3D crystal to lower temperatures than $[C_{10}]_2$ may suggest weaker side-chain ordering in the amphiphilic polymer.

Table 4. Comparison of DSC and GIWAXS Phase Transition Temperatures[1]

	DSC	GIWAXS[1]	DSC	GIWAXS
	$T_{m,\,peak}$ (°C)	T_m (°C)	$T_{c,\,peak}$ (°C)	T_c (°C)
$[C_{10}]_2$	154	149	138	141
$[C_{10}]_2$-co-$[OE_3]_2$	147	141	134	136
$[CH_3$-$C_{10}]$	219	214	204	208
$[CH_3$-$C_{10}]$-co-CH_3-$OE_3]$	220	218	189	195

1. GIWAXS temperatures are last temperature with clear diffraction T_m on heating and first temperature with clear diffraction on cooling.

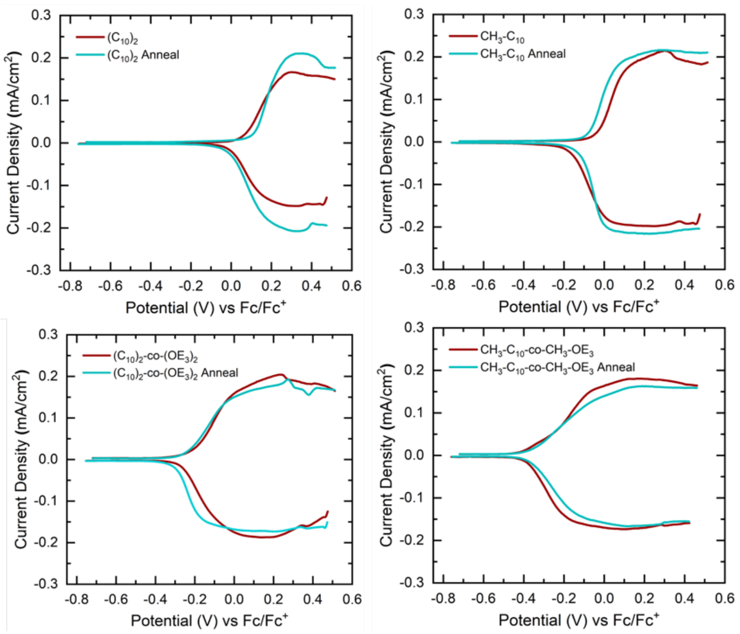

Onset of First Oxidation (V) vs Fc/Fc^+

Polymer	As Cast	Anneal
$[C_{10}]_2$	0.05	0.09
$[C_{10}]_2$-co-$[OE_3]_2$	-0.24	-0.26
CH_3-C_{10}	-0.05	-0.09
CH_3-C_{10}-co-CH_3-OE_3	-0.39	-0.39

Figure S30. Comparison between the first oxidation and reduction cycle using differential pulse voltammetry (DPV) for the as-cast and annealed PAcDOT films. The first redox cycle was used so that electrochemical break in did not impact the observed differences in oxidation behavior. Oxidation potential was determined by fitting a line to the initial onset of oxidation region and extrapolating to zero current. All electrochemistry was performed in 0.1 mol/L tetrabutylammonium hexafluorophosphate in propylene carbonate ($TBAPF_6$/PC) with a film coated on an indium tin oxide (ITO) working electrode, coiled Pt wire as a counter, and a Ag/AgCl pseudo-reference wire calibrated to the ferrocene/ferrocenium (Fc/Fc^+) redox couple ($E_{1/2}$ = 349 mV).

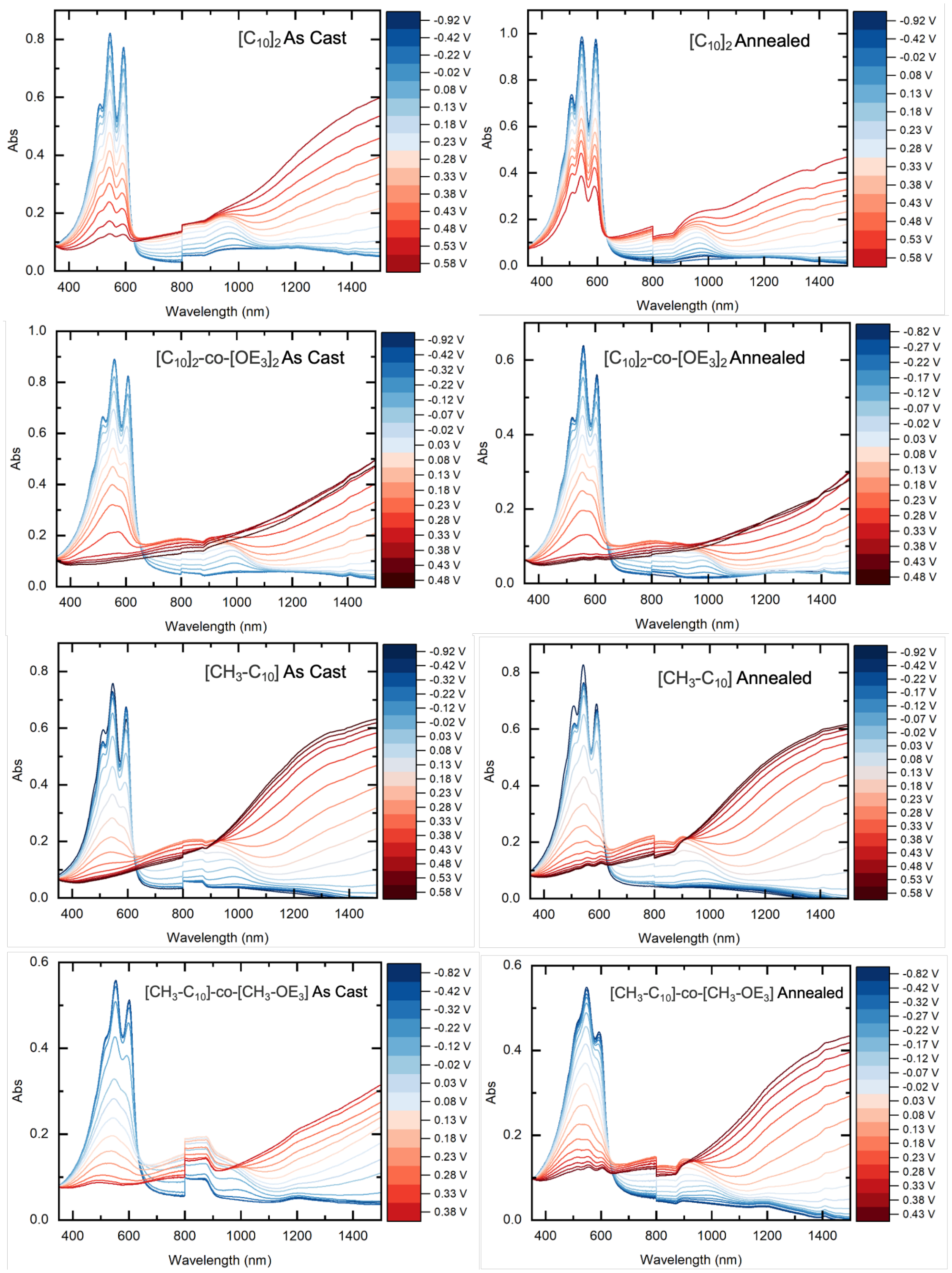

Figure S31. Spectroelectrochemistry plots for as-cast and annealed PAcDOT films. Performed in 0.1 mol/L $TBAPF_6$/PC with a film coated on an indium tin oxide (ITO) working electrode, coiled Pt wire as a counter, and a Ag/AgCl pseudo-reference wire calibrated to the Fc/Fc^+ redox couple ($E_{1/2}$ = 349 mV). Feature between (800 and 900) nm is an artifact from to the spectrometer.

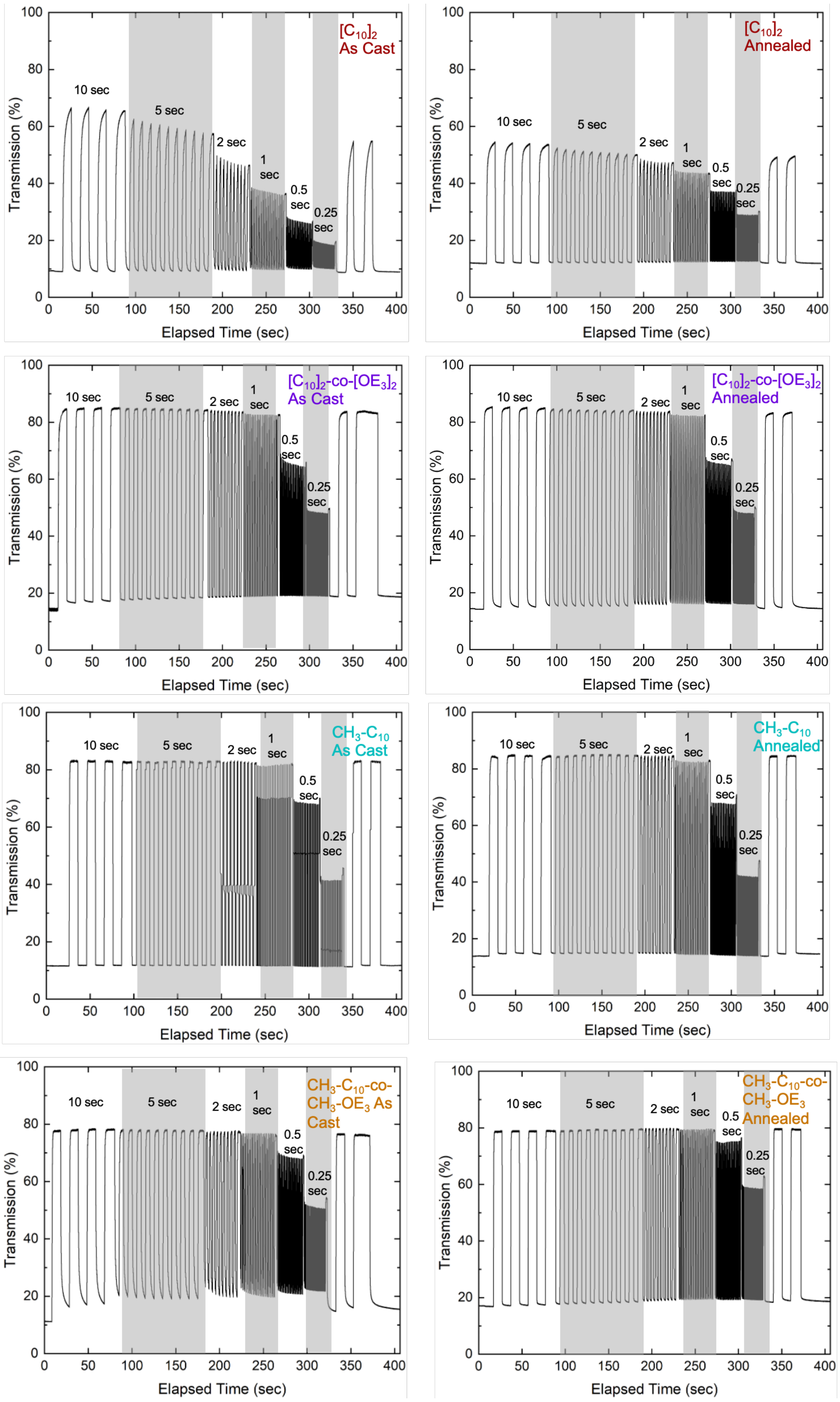

Figure S32. Chronoabsorptometry plots of PAcDOT films in 0.1 mol/L $TBAPF_6$ in propylene carbonate. The %T is measured as a function of redox pulse length. Contrast as a function of pulse length is extracted out of these raw plots by calculating $\%T_{bleach}$ - $\%T_{color}$ at the last full cycle of each given pulse length.

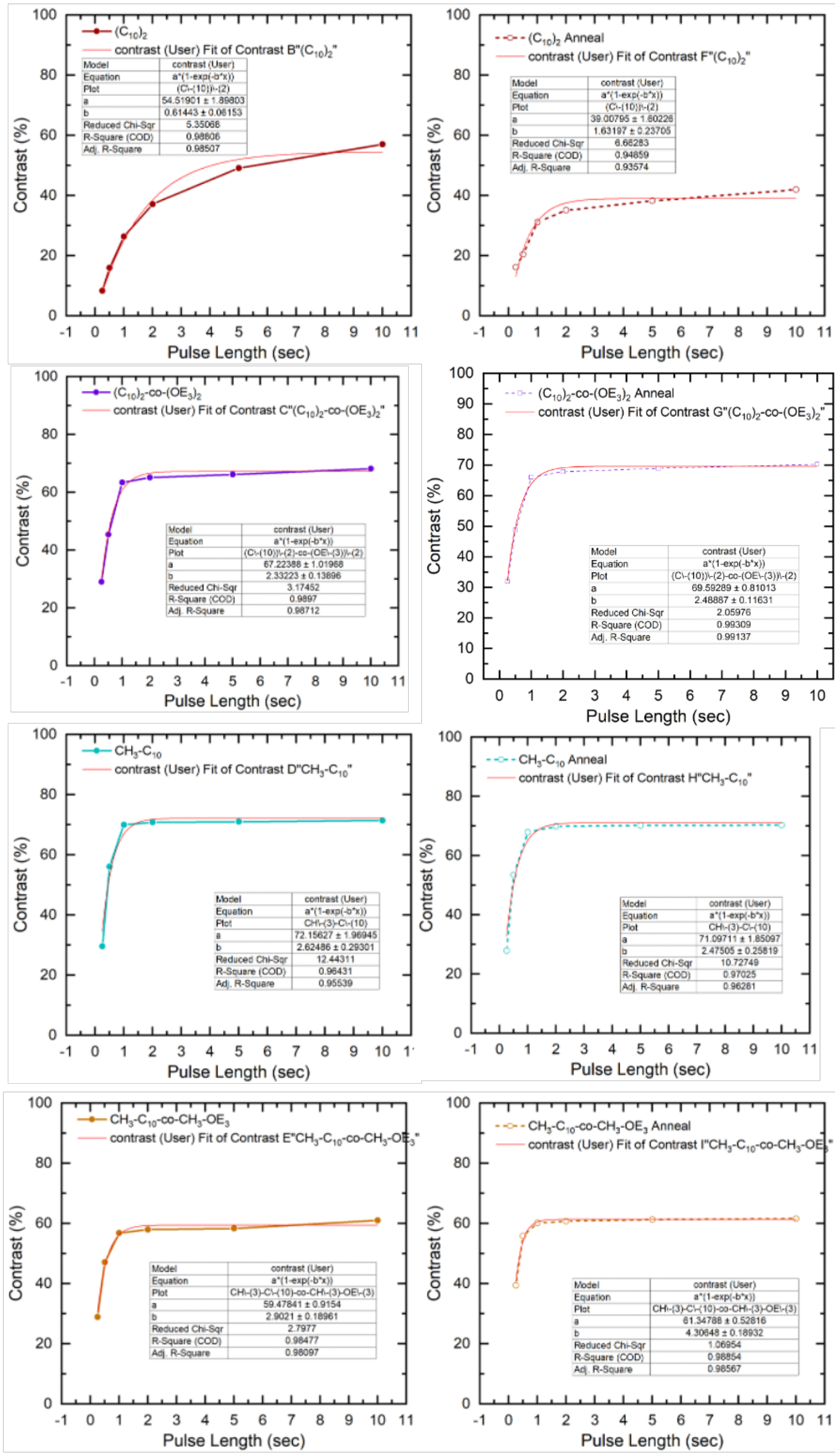

Figure S33. Fitting of contrast versus redox pulse length to $\Delta T(t) = \Delta T_{max}\left(1 - e^{-t/\tau}\right)$ as detailed in literature.[2] In the given plots, a = ΔT_{max} and b = $1/\tau$.

Table S5. Electrochemical Switching Time Constants for PAcDOT Films

PAcDOT	Film State	τ (s)
$[C_{10}]_2$	As Cast	1.63 ± 0.15
	Annealed	0.61 ± 0.08
$[C_{10}]_2$-co-$[OE_3]_2$	As Cast	0.43 ± 0.02
	Annealed	0.40 ± 0.02
$[CH_3-C_{10}]$	As Cast	0.38 ± 0.04
	Annealed	0.40 ± 0.05
$[CH_3-C_{10}]$-co-CH_3-OE_3	As Cast	0.34 ± 0.03
	Annealed	0.23 ± 0.01

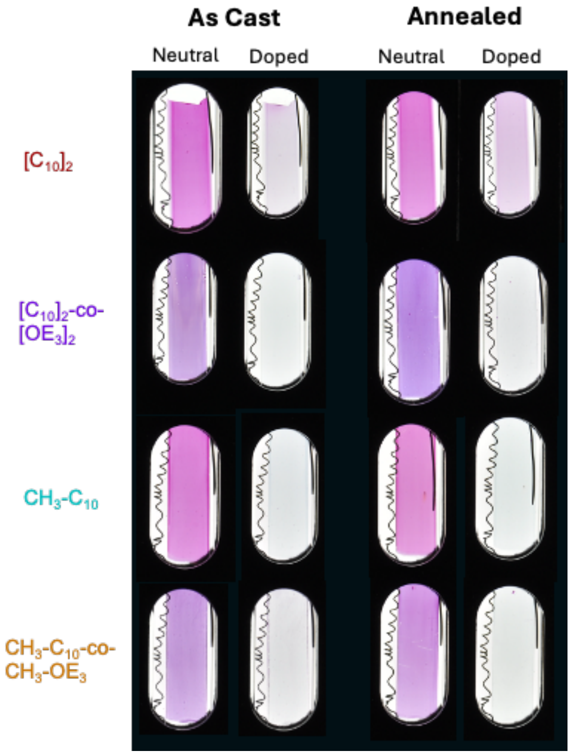

Figure S34. Photographs of the neutral and fully doped state (maximum potential of stability window) in 0.1 mol/L TBAPF6/PC.

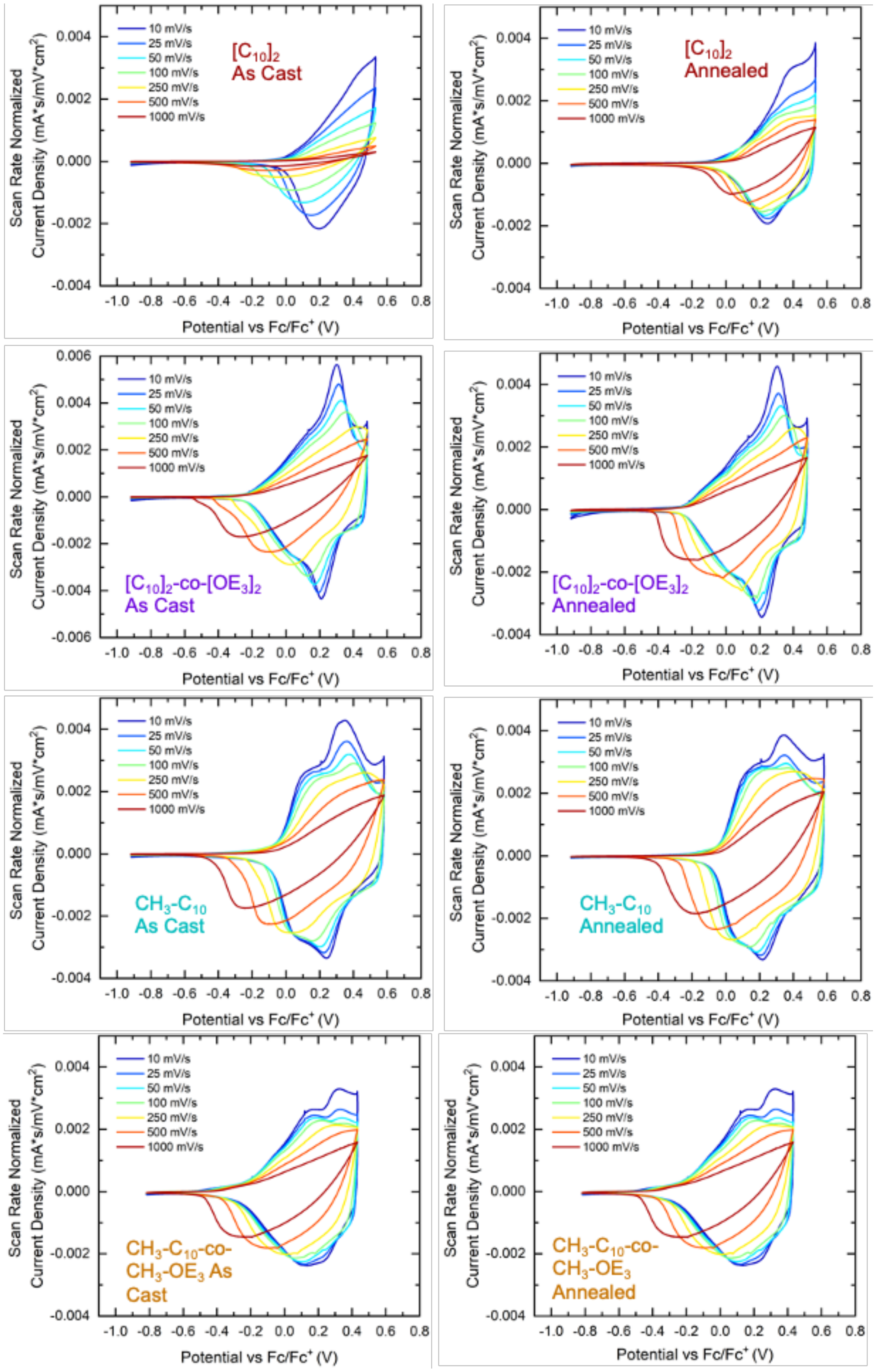

Figure S35. Scan rate dependent cyclic voltammetry (CV) data for all PAcDOTs. Current density is normalized by scan rate.

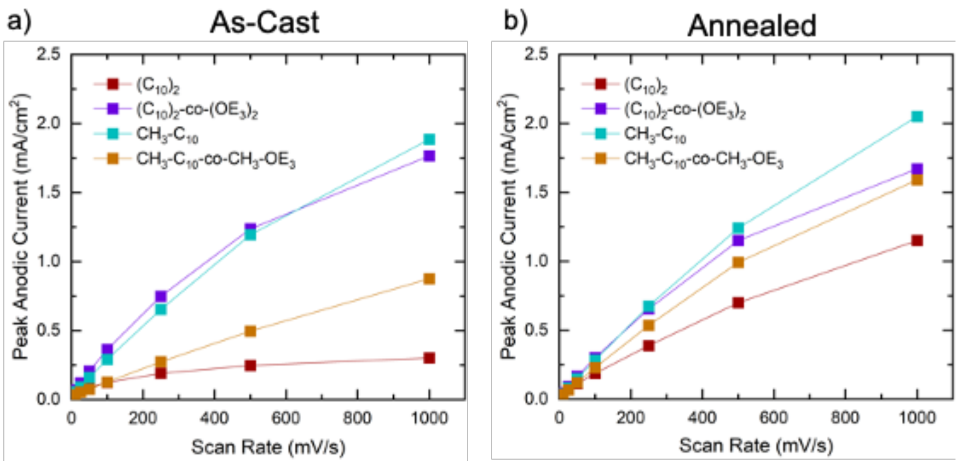

Figure S36. Peak current density as a function of scan rate for a) as-cast and b) annealed PAcDOT films. A linear relationship of peak anodic current out to higher CV scan rates indicates improved mixed transport kinetics.

Methods

Ex situ Grazing-Incidence Wide-Angle X-Ray Scattering

Films were processed onto clean, one-side polished silicon strips (500 µm, University Wafer). X-ray scattering measurements were carried out at Brookhaven National Lab at the Complex Material Scattering (CMS) beamline (11-ID). Film edges along the beam direction were gently removed by a cotton swap to eliminate edge effects from the coating process. Measurements were taken under vacuum. Typical film dimensions were 1 cm x 1 cm x 200 nm. Synchrotron radiation at 13.5 keV was exposed to the films for 10 s at 0.14° incident angle. The detector was a Pilatus 800k with a sample to detector distance of 258.6 mm. Data was worked up with the Nika package in Igor 9 and 2D image geometries corrected using the custom in-house code.[3, 4] Sector cuts were taken from 0° - 20° (in plane, Q_{xy}) and 80 °- 100 ° (out of plane, Q_z). Peaks were fit using Origin and Voight peak fitting. Peak deconvolution was done in the region between (1.3 to 1.7) $Å^{-1}$ by fitting three Voight peaks to the region and fixed the peak at 1.35 $Å^{-1}$ corresponding to

the amorphous halo. The other two peaks were allowed to vary their peak height and FWHM to optimize the fitting. Peak centers were used to calculate d-spacings according to $d_{hkl} = 2\pi/q$.

In situ Thermal Grazing-Incidence Wide-Angle X-Ray Scattering

In situ thermal GIWAXS was performed at the CMS beamline using the same configuration as ex situ measurements. A Linkam stage was used to heat and cool films at 5 °C/min from 30 °C to 160 °C or 230 °C, depending on the melting point of the polymer. Data was worked up with Nika.

Deconvolution of Variable Scan Rate DSC

Variable heating and cooling rate DSC experiments were performed on a DSC Discover by TA Instruments. Polymer powders were heated into the melt to erase thermal history. Samples were subsequently cooled at 10 °C/min, then heated at 20 °C/min, 10 °C/min, and 5 °C/min. High temperature phases upon heating were deconvoluted using two pseudo-Voigt peaks. Melting peak temperatures were used in the extrapolation of the zero-heating rate melting peak temperature. Enthalpies of each phase transition were then used to calculate the entropy of fusion.

Electrochemistry

For all electrochemical measurements, films were coated onto ITO glass slides (7 mm x 50 mm, 8-12 Ω/sq, Delta Technologies Ltd). Cyclic voltammetry was performed via EG&G PAR 273A potentiostat/galvanostat controlled by CorrWare. Potential was controlled via Ag/AgCl pseudo-reference wire ($E_{1/2}$ = 420 mV to Fc/Fc^+) and a coiled platinum wire was used as the counter electrode. 0.1 mol/L $TBAPF_6$ in propylene carbonate was used as the electrolyte. Unless otherwise indicated, all electrochemistry was performed on electrochemically broken in films, where multiple redox cycles were performed until there was no change in the onset of oxidation

or oxidative/reductive current. UV-Vis-NIR Spectroelectrochemistry was performed using this 3-electrode electrochemical cell in a quartz cuvette alongside an Agilent Technologies Cary 5000 UV-Vis-NIR Spectrophotometer. Chronoabsorptometry was performed with an Ocean Optics UV-Vis and Pine potentiostat controlled by Aftermath software. The absorption at λ_{max} was monitored by switching between the fully oxidized and fully reduced states at cycling rates of 10 s, 5 s, 2 s, 1 s, 0.5 s, and 0.25 s.

Cross-Polarized Microscopy and Circular Dichroism

Circular dichrosim spectra of the films and solutions were recorded using a JASCO 1500 spectrophotometer. To eliminate the effects of linear dichroism and birefringence, all samples were examined by averaging four measurements, incorporating 90° in-plane and 180° out-of-plane rotations.[5] The birefringence of the solutions, thin films, and powders was characterized using cross-polarized optical microscopy (Nikon Eclipse Ci-POL). A Linkam stage was used to heat/cool the thin film/polymer powder.

Additional Note:

Certain equipment, instruments, software, or materials are identified in this paper in order to specify the experimental procedure adequately. Such identification is not intended to imply recommendation or endorsement of any product or service by NIST, nor is it intended to imply that the materials or equipment identified are necessarily the best available for the purpose.

References

(1) Snyder, C. R.; Kline, R. J.; DeLongchamp, D. M.; Nieuwendaal, R. C.; Richter, L. J.; Heeney, M.; McCulloch, I., Classification of semiconducting polymeric mesophases to optimize device postprocessing. *Journal of Polymer Science Part B: Polymer Physics* **2015,** *53* (23), 1641-1653.

(2) Hassab, S.; Shen, D. E.; Österholm, A. M.; Da Rocha, M.; Song, G.; Alesanco, Y.; Viñuales, A.; Rougier, A.; Reynolds, J. R.; Padilla, J., A new standard method to calculate electrochromic switching time. *Solar Energy Materials and Solar Cells* **2018,** *185*, 54-60.

(3) Baker, J. L.; Jimison, L. H.; Mannsfeld, S.; Volkman, S.; Yin, S.; Subramanian, V.; Salleo, A.; Alivisatos, A. P.; Toney, M. F., Quantification of thin film crystallographic orientation using X-ray diffraction with an area detector. *Langmuir* **2010,** *26* (11), 9146-9151.

(4) Ilavsky, J., Nika: software for two-dimensional data reduction. *Journal of Applied Crystallography* **2012,** *45* (2), 324-328.

(5) Yao, Y.; Ugras, T. J.; Meyer, T.; Dykes, M.; Wang, D.; Arbe, A.; Bals, S.; Kahr, B.; Robinson, R. D., Extracting Pure Circular Dichroism from Hierarchically Structured CdS Magic Cluster Films. *ACS Nano* **2022,** *16* (12), 20457-20469.
